# Lysosomal dysfunction and impaired autophagy underlie the pathogenesis of amyloidogenic light chain-mediated cardiotoxicity

**DOI:** 10.15252/emmm.201404190

**Published:** 2014-10-15

**Authors:** Jian Guan, Shikha Mishra, Yiling Qiu, Jianru Shi, Kyle Trudeau, Guy Las, Marc Liesa, Orian S Shirihai, Lawreen H Connors, David C Seldin, Rodney H Falk, Calum A MacRae, Ronglih Liao

**Affiliations:** 1 Division of Cardiovascular Medicine, Brigham and Women's Hospital, Harvard Medical SchoolBoston, MA, USA; 2 Department of Medicine, Boston University School of MedicineBoston, MA, USA; 3 Amyloidosis Center, Boston University School of MedicineBoston, MA, USA; 4 Cardiac Amyloidosis Program, Brigham and Women's Hospital, Harvard Medical SchoolBoston, MA, USA

**Keywords:** amyloidosis, autophagy, cardiac toxicity, lysosome, mitochondria

## Abstract

AL amyloidosis is the consequence of clonal production of amyloidogenic immunoglobulin light chain (LC) proteins, often resulting in a rapidly progressive and fatal amyloid cardiomyopathy. Recent work has found that amyloidogenic LC directly initiate a cardio-toxic response underlying the pathogenesis of the cardiomyopathy; however, the mechanisms that contribute to this proteotoxicity remain unknown. Using human amyloidogenic LC isolated from patients with amyloid cardiomyopathy, we reveal that dysregulation of autophagic flux is critical for mediating amyloidogenic LC proteotoxicity. Restoration of autophagic flux by pharmacological intervention using rapamycin protected against amyloidogenic light chain protein-induced pathologies including contractile dysfunction and cell death at the cellular and organ level and also prolonged survival in an *in vivo* zebrafish model of amyloid cardiotoxicity. Mechanistically, we identify impaired lysosomal function to be the major cause of defective autophagy and amyloidogenic LC-induced proteotoxicity. Collectively, these findings detail the downstream molecular mechanisms underlying AL amyloid cardiomyopathy and highlight potential targeting of autophagy and lysosomal dysfunction in patients with amyloid cardiomyopathy.

## Introduction

AL or light chain amyloidosis (formerly known as primary amyloidosis) is the most commonly diagnosed systemic amyloidosis in the United States and Europe (Merlini *et al*, 2011), in which widespread tissue infiltration and deposition of amyloid fibrils derived from clonal immunoglobulin light chain (LC) proteins causes multi-organ dysfunction. Greater than 70% of patients with primary LC amyloidosis present with cardiac involvement (Madan *et al*, 2010; Falk, 2011), which can progress to debilitating heart failure symptoms and early cardiovascular death (Falk, 2005; Falk, 2011). To date, there are no targeted treatments for amyloid cardiomyopathy (Falk, 2011), owing to a lack of understanding of the basic mechanisms that underlie the pathogenesis of the disease. While amyloid fibril deposition within the heart has long been hypothesized to be responsible for disease pathophysiology, there often is dissociation between the degree of amyloid fibril deposition and cardiovascular outcomes. We and others have found that circulating amyloidogenic light chain proteins (AL-LC) directly initiate a potent cardiotoxic effect, independent of fibril deposition, and this cardiotoxicity is critical to manifestations of amyloid cardiomyopathy, both *in vitro* and *in vivo* (Liao *et al*, 2001; Brenner *et al*, 2004; Migrino *et al*, 2010, 2011; Shi *et al*, 2010; Sikkink & Ramirez-Alvarado, 2010; Shin *et al*, 2012). While these findings have changed our understanding of AL amyloid cardiomyopathy, from one of just passive fibril infiltration to also acknowledging a direct proteotoxicity, the basic mechanisms by which this proteotoxicity results in cardiomyopathy remain unknown. Furthermore, an increase in oxidative stress and reactive oxygen species (ROS) production is one of the consequences associated with AL-mediated proteotoxicity. However, the source for this increased ROS is unknown.

A growing body of evidence demonstrates that mitochondrial quality control alterations contribute and can be central to a number of human diseases including Alzheimer's, Parkinson's, Huntington's, diabetes and cardiovascular disease (Harris & Rubinsztein, 2012; Nixon, 2013). The heart is particularly sensitive to perturbations of mitochondrial function, given the energetic requirements of contractile function. Removal of damaged mitochondria is essential to prevent increased ROS generation, decreased ATP production and loss of cellular function. Damaged mitochondria are cleared intracellularly by a complex quality control mechanism involving mitophagy and the lysosome. Mitophagy refers to a macro-autophagic process that selectively removes mitochondria. Of note, macro-autophagy has also been implicated in handling proteotoxic events that cannot be mediated by the proteasome. Herein, utilizing *in vitro* isolated cardiomyocytes and an *in vivo* zebrafish model of AL-LC toxicity, we find that disruption of autophagic flux is the underlying mechanism critical for the induction of mitochondrial dysfunction and development of AL amyloid cardiomyopathy.

## Results

### AL-LC triggers mitochondrial dysfunction and ROS production

**Figure 1:**
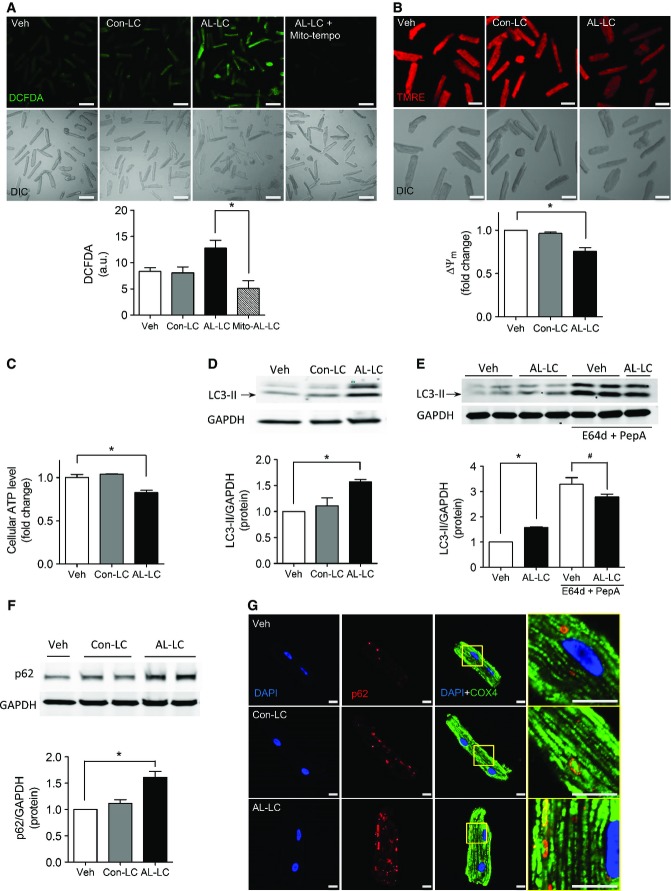
AL-LC causes mitochondrial dysfunction and autophagy dysregulation *in vitro* Using the fluorescent indicator DCFDA, ROS was measured in isolated cardiomyocytes treated with vehicle, Con-LC, AL-LC or AL-LC + Mito-TEMPO. Representative fluorescent and bright field images are shown in the top panels, and quantitative analysis is summarized in the graph below. AL-LC but not Con-LC increased ROS and this was blocked by Mito-TEMPO, indicating that ROS is derived from mitochondria. Scale bar = 100 μm. *N* = 3. *P = 0.020.Mitochondrial membrane potential was measured using TMRE fluorescent dye in cardiomyocytes following 24-h treatment with vehicle, Con-LC or AL-LC. Representative TMRE fluorescent and bright field microscopy images are shown in the top panels, and TMRE fluorescence signal was quantified and summarized below. AL-LC exposure resulted in loss of mitochondrial membrane potential compared to the other groups. Scale bar = 50 μm. *N* = 3. *P = 0.031.Quantitative summary of cellular ATP levels in cardiomyocytes following 24-h exposure to vehicle, Con-LC or AL-LC. *N* = 3. *P = 0.020.Immunoblot analysis of LC3-II expression in cardiomyocytes following 24-h exposure to vehicle, Con-LC or AL-LC. GAPDH was used as a loading control. Quantitative results are shown in the graph below for comparison. LC3-II expression was significantly increased in the AL-LC group compared to vehicle and Con-LC. *N* = 3. *P = 1.9 × 10^−6^.Immunoblot analysis of AL-LC-induced LC3-II expression in the presence of lysosomal inhibitors E64d and Pepstatin A. GAPDH is used as a loading control. Quantitative results in the graph below show that LC3-II levels in the AL-LC group do not exceed vehicle-treated LC3-II levels following lysosomal inhibition, indicating minimal perturbation to initiation. *N* = 6. *P = 1.4 × 10^−6^, ^#^*P* = 0.041.Immunoblot and quantitative analysis of p62 expression in cardiomyocytes following 24-h exposure to vehicle, Con-LC or AL-LC. Results show a decrease in autophagic flux seen by increased p62 accumulation in the AL-LC group. *N* = 3. *P = 0.033.Confocal imaging of immunofluorescence staining of p62 (red) in cardiomyocytes following 24-h exposure to vehicle, Con-LC or AL-LC. Mitochondrial co-localization was visualized using mitochondrial protein COX4 (green) co-staining, and nuclear staining with DAPI (blue). Scale bar = 10 μm. Source data are available online for this figure.

We have shown that human AL-LC protein provokes excessive ROS production and subsequent cellular dysfunction and cell death in isolated cardiomyocytes (Brenner *et al*, 2004; Shi *et al*, 2010); however, the source of ROS production has yet to be identified. To determine whether mitochondria contribute to AL-LC-induced ROS production, isolated cardiomyocytes were pretreated with the mitochondrial-targeted ROS scavenger, Mito-TEMPO, and exposed to human AL-LC. Using the ROS-sensitive fluorescent dye, DCFDA, we found that increased AL-LC-elicited ROS was abolished with Mito-TEMPO to levels comparable to cells treated with vehicle (Veh) or control light chain (Con-LC) proteins isolated from patients with multiple myeloma (Fig 1A). In addition, AL-LC treatment of cardiomyocytes was associated with decreased mitochondrial membrane potential (depolarization) as determined by TMRE (Fig 1B). One process that could explain this depolarization is decreased mitochondrial bioenergetics function associated with decreased ATP synthesis. In order to confirm this, we measured cellular ATP levels, as mitochondria are the main contributor to ATP levels in cardiomyocytes. Total ATP levels were decreased by AL-LC and not by addition of Con-LC (Fig 1C). Thus, these data collectively demonstrate mitochondrial dysfunction and increased mitochondrial ROS production caused by AL-LC in cardiomyocytes.

### AL-LC impairs autophagic flux

Defective mitochondria are cleared intracellularly by a complex macro-autophagic response (Codogno, 2014). By Western blot, levels of the autophagy marker LC3-II were markedly increased in cardiomyocytes exposed to AL-LC (Fig 1D), as well as the number of autophagosomes in AL-LC-exposed cardiomyocytes overexpressing GFP-LC3 (Mizushima *et al*, 2010), (Supplementary Fig S1). Increased LC3-II levels and number of autophagosomes (detected as GFP-LC3 punctae) may be indicative of either elevated autophagy induction or defective clearance. To distinguish between induction and clearance of autophagosomes, E64d and Pepstatin A were used to inhibit lysosomal enzymes and impede autophagosome clearance. LC3-II levels accumulated less after lysosome inhibition in AL-LC cardiomyocytes, indicative of a decrease in autophagosome clearance. This decrease was explained both by increased basal LC3-II levels and by decreased LC3-II levels after E64D and Pepstatin A treatments in AL-LC cardiomyocytes when compared to control (Fig 1E). Furthermore, AL-LC resulted in an increase in p62 accumulation, an established marker of autophagic clearance (Fig 1F). We next addressed whether this alteration in macroautophagy was also associated with decreased mitophagic clearance. Immunofluorescent staining of isolated adult cardiomyocytes exposed to AL-LC showed an increase in p62 levels co-localized with mitochondria, suggesting a perturbation in mitophagy (Fig 1G). Taken together, our data suggest a defect in autophagy flux in cardiomyocytes subjected to human AL-LC protein, with a corresponding inhibition of mitochondrial clearance.

### Restoration of autophagic flux attenuates AL-LC-induced cellular dysfunction and cell death *in vitro*

**Figure 2:**
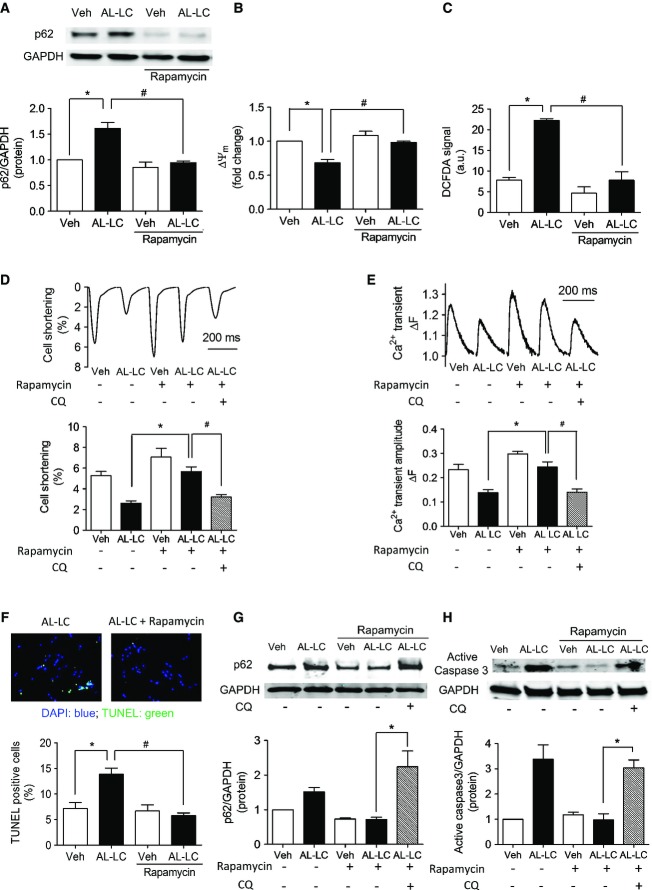
Restoration of autophagic flux with rapamycin attenuates AL-LC-induced cellular dysfunction and cell death *in vitro* Immunoblot analysis of p62 on cardiomyocytes following 24-h exposure to vehicle, Con-LC or AL-LC in the absence or presence of 10 nM rapamycin. Quantitative results summarized below show decreased p62 expression following rapamycin treatment. *N* = 3. *P = 0.003, ^#^*P* = 0.006.Mitochondrial function is rescued by rapamycin treatment, shown by quantitative analysis of mitochondrial membrane potential using TMRE dye in cardiomyocytes following exposure to vehicle, Con-LC or AL-LC for 24 h in the presence or absence of rapamycin. *N* = 3. *P = 0.046, ^#^*P* = 0.005 between indicated groups.ROS levels are reduced by treatment with rapamycin, shown using DCFDA in cardiomyocytes exposed to vehicle, Con-LC or AL-LC. *N* = 3. *P = 2.3 × 10^−4^, ^#^*P* = 0.002 between indicated groups.Contractile function was measured in cardiomyocytes exposed to AL-LC for 24 h in the absence or presence of rapamycin with or without chloroquine (2.5 μM). Quantitative analysis was performed by calculating percent cell shortening. *N* = 3. *P = 3.3 × 10^−4^, ^#^*P* = 0.007.Calcium transient amplitude, measured in isolated cardiomyocytes, was quantified following exposure to vehicle or AL-LC in the presence or absence of rapamycin with or without chloroquine. Representative tracings are shown, and quantitative analysis in the graph shows a rescue of AL-LC-induced decrease in calcium transient amplitude following rapamycin treatment. *N* = 3, *P = 0.002, ^#^*P* = 0.006.Rapamycin treatment reduces apoptosis in cardiomyocytes exposed to AL-LC. TUNEL staining was performed to quantify cell death in cardiomyocytes following exposure to vehicle, Con-LC or AL-LC with or without rapamycin treatment. Cell death was measured as percent TUNEL-positive nuclei relative to total cell number. *N* = 3. *P = 0.015, ^#^*P* = 0.035 between indicated groups.Verification that rapamycin rescue was autophagy dependent was demonstrated using chloroquine (2.5 μM) administered to AL-LC + rapamycin-treated cardiomyocytes. p62 accumulation was measured using immunoblot analysis, with GAPDH as a loading control. *N* = 5. *P = 0.023.Cardiomyocytes were treated with chloroquine in the presence of rapamycin, and immunoblot analysis was performed to probe for active caspase 3 levels, normalized to GAPDH expression. *N* = 3. *P = 0.007. Source data are available online for this figure.

To determine whether autophagy dysregulation was causal for AL-LC-induced cardiotoxicity, rapamycin, an inhibitor of mTOR signaling and a potent enhancer of both autophagosome formation and clearance, was used to restore autophagic flux in cardiomyocytes exposed to AL-LC. Restoration of autophagosome clearance by rapamycin was confirmed by significant reduction in p62 accumulation in cardiomyocytes exposed to AL-LC (Fig 2A). Concomitant with decreased p62 levels, rapamycin-treated cardiomyocytes showed significant attenuation of both mitochondrial dysfunction and intracellular ROS levels (Fig 2B and C), and protection against AL-LC-induced cellular contractile dysfunction (Fig 2D) and concomitant impaired intracellular calcium homeostasis (Fig 2E) as well as cell death (Fig 2F). Importantly, the concentration of rapamycin used (10 nM) (Dehay *et al*, 2010) did not affect downstream substrates of mTOR as measured by S6 kinase activation (Supplementary Fig S2). In addition, pharmacologic inhibition of autophagy by chloroquine (CQ) reversed rapamycin attenuation of p62 accumulation in cardiomyocytes exposed to AL-LC (Fig 2G) and abrogated the beneficial effects of rapamycin on contractile function (Fig 2D), calcium transient amplitude (Fig 2F) and cell survival (Fig 2H), consistent with rapamycin protecting against AL-LC via improvement of autophagic flux.

### Rapamycin protects against AL-LC proteotoxicity *in vivo*

**Figure 3:**
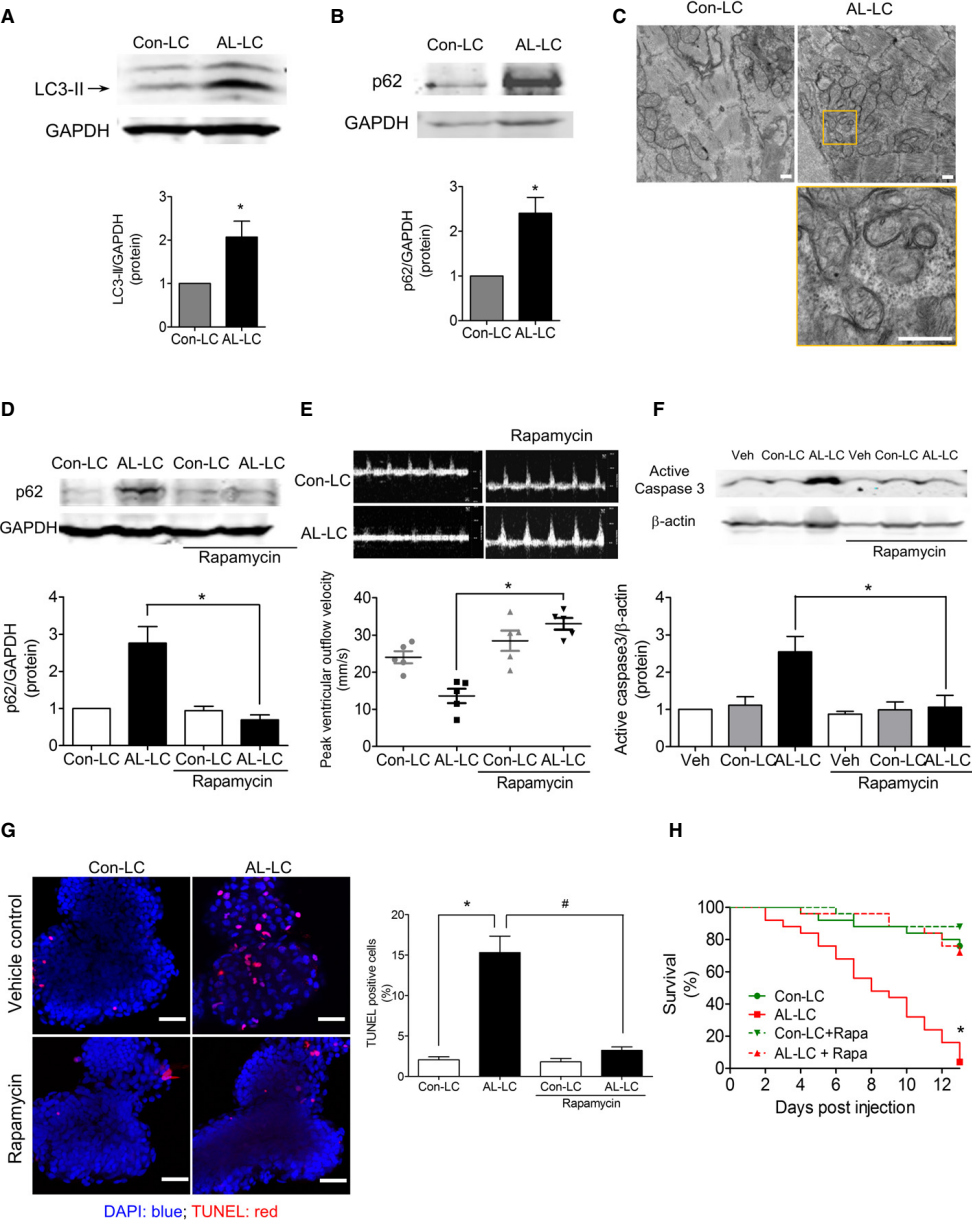
Restoration of autophagic flux via rapamycin attenuates AL-LC-induced cellular dysfunction and cell death *in vivo* A, B Immunoblot analysis of LC3-II and p62 expression in zebrafish lysate, 3 days post-injection of Con-LC or AL-LC (100 μg/ml). GAPDH was used as a loading control. *N* = 5 (A); *N* = 4 (B). *P = 0.043, ^#^*P* = 0.029. C Transmission electron micrographs of cardiac tissue of Con-LC and AL-LC-injected zebrafish 3 days post-injection (5 days post-fertilization). Top panels show changes in mitochondrial morphology and presence of double-membrane structures (autophagosomes) at 6,800× in AL-LC fish compared to Con-LC fish. Bottom panel shows higher magnification of mitochondrial structure and autophagosomes. Scale bar = 500 nm. *N* = 3 per group. D Zebrafish were treated with 10 nM rapamycin after receiving Con-LC or AL-LC injection. Immunoblot analysis was performed to measure p62 expression and normalized to GAPDH as a loading control. Fifteen fish were homogenized per experiment. Quantitative analysis is shown for comparison below representative blots. *N* = 3. *P = 0.011. E Peak flow was measured in zebrafish embryos using color Doppler echocardiography. Representative color Doppler peak flow tracings of zebrafish 3 days post-injection with or without treatment with rapamycin. Peak flow quantitation analysis is shown in the panel below. *N* = 5. *P = 0.001. F Cell death was quantified in zebrafish 3 days post-injection with or without rapamycin treatment. Expression of active caspase 3 was measured using immunoblot. 15 zebrafish were homogenized per experiment. *N* = 4. *P = 0.029. G Representative confocal images of hearts isolated from zebrafish 3 days post-injection with or without rapamycin treatment. Hearts are stained for TUNEL-positive nuclei (red) and DAPI counterstain. TUNEL-positive nuclei are quantified and graphed in the right panel. Scale bar = 25 μm. *N* = 4 per group. *P = 6.7 × 10^−4^, ^#^*P* = 0.001. H Kaplan–Meier analysis of zebrafish survival following injection of Con-LC or AL-LC in the absence or presence of rapamycin. Survival was monitored daily. The survival of AL-LC-injected fish was significantly prolonged in the presence of rapamycin. *N* =  25 per group. *P = 0.0001. Source data are available online for this figure.

To determine the role of AL-LC-induced impaired autophagy *in vivo*, we utilized a recently reported zebrafish model of AL-LC cardiotoxicity, characterized by impaired cardiac function and early cardiovascular death following injection of human AL-LC (Mishra *et al*, 2013). Consistent with our *in vitro* findings, zebrafish injected with AL-LC showed increased LC3-II and p62 levels (Fig 3A–B) compared to Con-LC. Electron microscopy of heart tissue revealed increased autophagosome number as indicated by the accumulation of double-membrane vesicle structures with AL-LC exposure (Fig 3C). Autophagic flux was restored in AL-LC-injected zebrafish via treatment with 10 nM rapamycin (Tobin & Beales, 2008), seen by decreased p62 comparable to control levels (Fig 3D). Peak aortic flow, an indicator of cardiac function, was decreased in AL-LC-injected fish (Fig 3E) and restored to control levels with rapamycin treatment. Similarly, AL-LC-triggered cell death in zebrafish hearts was reduced following rapamycin treatment (Fig 3F and G). Survival was markedly impaired following injection of human AL-LC in zebrafish and was significantly rescued with rapamycin treatment (Fig 3H). Rapamycin did not alter survival in Con-LC animals (Fig 3H). Together, our *in vivo* data provide further evidence for the central role of autophagic dysfunction in the pathogenesis of amyloid cardiotoxicity and highlight the use of rapamycin as a potential therapeutic approach for treatment of this disease.

### Lysosomal dysfunction directly contributes to AL-LC-triggered impaired autophagy

**Figure 4:**
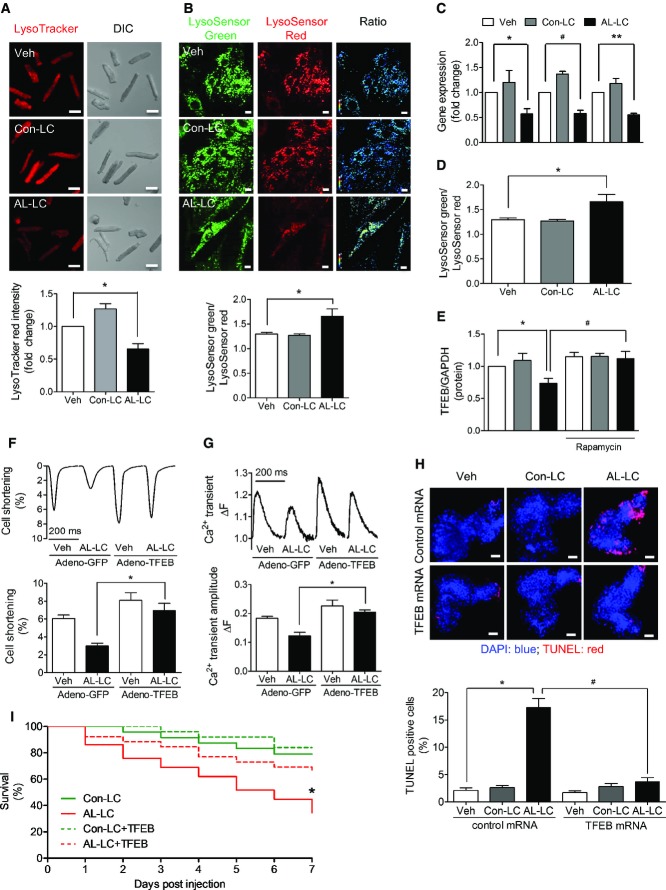
Lysosomal dysfunction contributes to AL-LC-induced dysregulation of autophagy and consequent cellular dysfunction and death *in vitro* and *in vivo* Lysosomal labeling using LysoTracker red in cardiomyocytes following exposure to vehicle, Con-LC or AL-LC is shown (top panels), with corresponding bright field images shown below. Quantitation of fluorescent signal is shown in the graph on the right. Scale bar = 50 μm. *N* = 6. *P = 0.009.Alterations in lysosomal pH were measured using LysoSensor in cardiomyocytes treated with vehicle, Con-LC or AL-LC. pH changes were measured by calculating the ratio between green (basic) and red (acidic) LysoSensor signal. Quantitation is shown on the right indicating loss of lysosomal acidity in AL-LC treated cardiomyocytes. Scale bar = 10 μm. *N* = 8. *P = 0.038.Quantitative PCR analysis of cardiomyocytes following 24-h exposure to vehicle, Con-LC or AL-LC reveals changes in mRNA encoding the lysosomal gene products cathepsin D and vacuolar ATPase subunits 1 and 2. All three targets were significantly downregulated in the AL-LC group. *N* = 3. *P = 0.015, ^#^*P* = 0.002, **P = 1.9 × 10^−4^.Quantitative PCR analysis of cardiomyocytes following 24-h exposure to vehicle, Con-LC and AL-LC reveals decreased mRNA level of the lysosomal transcriptional factor TFEB. *N* = 4. *P = 0.003.Protein expression of TFEB in cardiomyocytes was measured using immunoblot analysis following 24-h exposure to vehicle, Con-LC or AL-LC. AL-LC-induced downregulation of TFEB protein expression was prevented by rapamycin treatment. *N* = 3. *P = 0.043, ^#^*P* = 0.032.TFEB was overexpressed in cardiomyocytes using adenovirus, and adenoviral GFP overexpression was used as a control. Contractile function was measured following exposure to vehicle or AL-LC. In cells overexpressing TFEB, AL-LC-induced decreased cell shortening was rescued compared to GFP-expressing myocytes. *N* = 5. *P = 0.002.TFEB was overexpressed in cardiomyocytes using adenovirus. Adeno-GFP was used as a control. Calcium transient amplitude was measured following exposure to either vehicle or AL-LC. In cells overexpressing TFEB, AL-LC-induced decrease in calcium amplitude was rescued compared to control groups. *N* = 5. *P = 0.002.Hearts were isolated 2 days post-injection of vehicle, Con-LC or AL-LC from zebrafish overexpressing TFEB, or control mRNA. Hearts were stained for TUNEL-labeled nuclei with a DAPI counterstain. Cell death was calculated as a percent of TUNEL-positive nuclei to total cell number. Scale bar = 20 μm. *N* = 3–5 per group. *P = 0.003, ^#^*P* = 0.002.Kaplan–Meier analysis of survival following injection of Con-LC or AL-LC in zebrafish overexpressing control or TFEB mRNA. Survival was monitored daily. During the time course of transient overexpression of TFEB, AL-LC-induced mortality was rescued significantly. *N* =  25 per group. *P = 0.0003.

Our results suggest that AL-LC-induced dysregulation of autophagic flux may reside at the stage of autophagosome clearance, the final step in the autophagy process in which the lysosome plays a pivotal role. We sought to examine lysosomal function in response to AL-LC exposure. The number of acidic vesicles (including lysosomes) per cell, as measured using LysoTracker staining, was markedly compromised in isolated cardiomyocytes exposed to AL-LC for 24 h (Fig 4A), concomitant with a loss of lysosomal acidity, assessed by LysoSensor, a pH-sensitive fluorescent probe (Fig 4B) 24 h following AL-LC exposure. Associated with loss of lysosomal function, quantitative PCR revealed downregulation of lysosome-related genes including cathepsin D, lysosomal-specific vacuolar ATPases (Fig 4C), as well as a transcriptional regulator of lysosomal biogenesis and function, TFEB (transcription factor EB) at the mRNA (Fig 4D) and protein (Fig 4E) levels following 24 h of AL-LC exposure. Importantly, decreased TFEB expression was restored to baseline levels following rapamycin treatment (Fig 4E).

To determine whether downregulation of TFEB is central to AL-LC-induced cardiotoxicity, TFEB was overexpressed in isolated cardiomyocytes (Supplementary Fig S3A) and in zebrafish (Supplementary Fig S3B). Overexpression of TFEB protected against contractile dysfunction and restored calcium transient amplitude in cardiomyocytes exposed to AL-LC (Fig 4F–G) and prevented AL-LC-associated cardiac cell death *in vivo* (Fig 4H) with greatly improved survival (Fig 4I).

**Figure 5:**
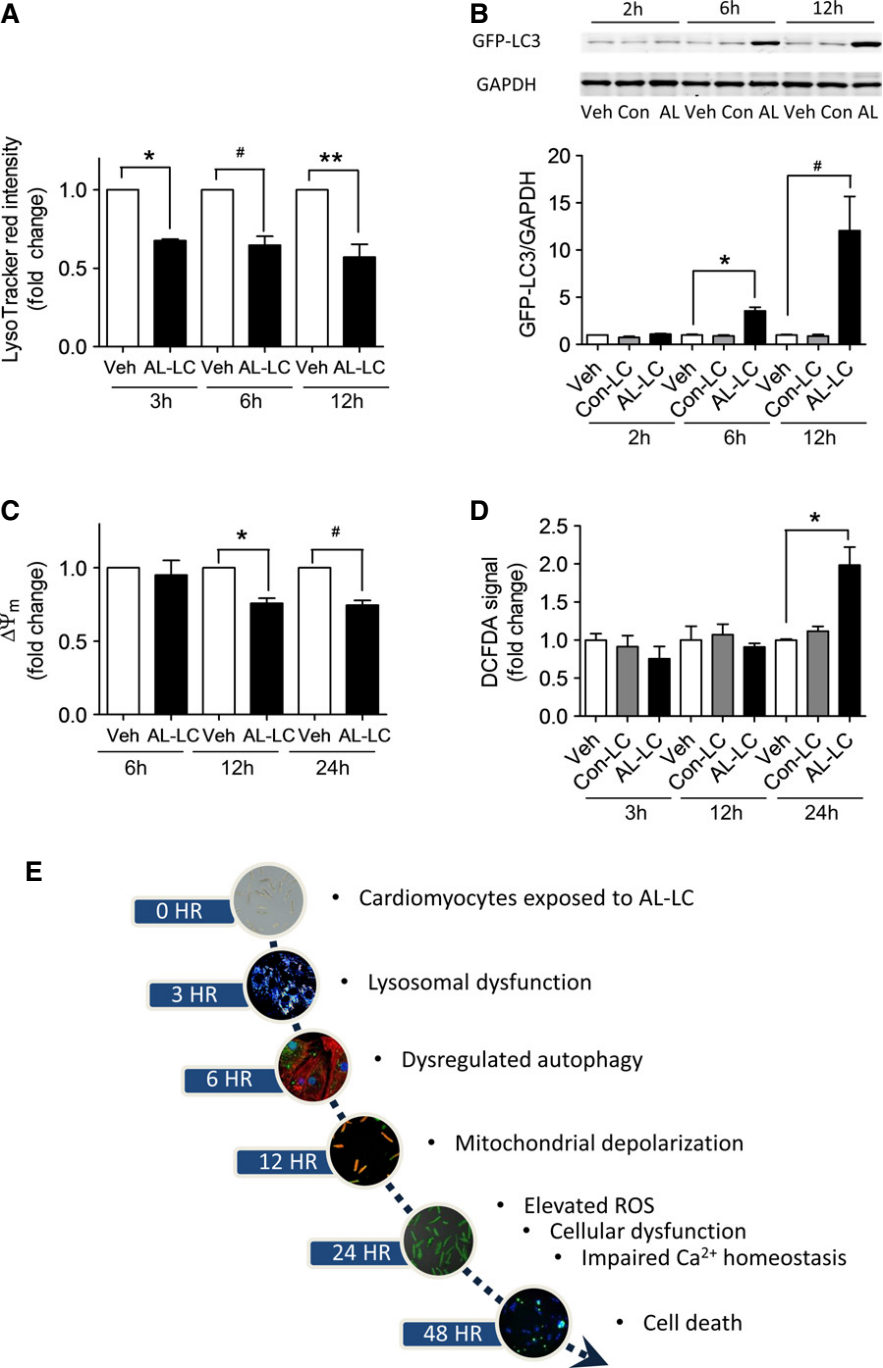
Temporal analysis of AL-LC-induced toxicity and dysregulation of autophagy Lysosomal labeling in cardiomyocytes following exposure to vehicle, Con-LC or AL-LC using LysoTracker red at 3, 6 and 12 h post-AL-LC exposure. Quantitation of fluorescent signal is shown in the graph. *N* = 3. *P = 0.011, ^#^*P* = 0.032, **P = 0.029.Dysregulation of autophagy following AL-LC exposure was monitored using a GFP-LC3 cleavage assay. Degradation of the GFP-LC3 fusion protein was monitored via immunoblotting against GFP antibody at 2, 6, and 12 h post-AL-LC exposure. A significant delay in the degradation of GFP-LC fusion protein was seen by accumulation of GFP-LC fusion protein in cardiomyocytes starting 6 h following exposure to AL-LC. *N* = 5. *P = 0.003, ^#^*P* = 0.038.Mitochondrial membrane potential was measured using TMRE fluorescent dye in cardiomyocytes 6, 12 and 24 h following treatment with vehicle or AL-LC. TMRE fluorescence signal was quantified and summarized in the graph. AL-LC exposure resulted in loss of mitochondrial membrane potential compared to the other groups at 12 and 24 h post-AL-LC exposure. *N* = 3. *P = 0.020, ^#^*P* = 0.029.Using fluorescent indicator DCFDA, ROS was measured in isolated cardiomyocytes treated with vehicle, Con-LC and AL-LC for 3, 12 and 24 h. Quantitative analysis is summarized in the graph. AL-LC but not Con-LC increased ROS only at 24 h following AL-LC exposure. *N* = 3. *P = 0.004.Schematic illustration of temporal events involved in AL-LC-induced pathology. Source data are available online for this figure.

To determine the temporal importance of lysosomal and autophagic dysfunction, we examined the time course of activation of previously established critical components of the AL-LC cardiotoxic response. We found that lysosomal function was impaired early, within 3 h of AL-LC exposure in isolated cardiomyocytes (Fig 5A). Six hours following AL-LC exposure, autophagic dysfunction was noted by accumulation of GFP-LC3, a result of reduced autophagic degradation (Ni *et al*, 2011) (Fig 5B). Loss of mitochondrial clearance was followed by decreased mitochondrial membrane potential, as measured by the mitochondrial membrane potential-sensitive dye TMRE (Fig 5C) at 12 and 24 h following AL-LC exposure, respectively. Increased ROS was detected by DCFDA (Fig 5D) 24 h following AL-LC exposure indicating it to be a late event. The temporal cascade of molecular events triggered by the AL-LC is summarized in Fig 5E.

### Human AL amyloid cardiomyopathy is associated with impaired autophagy and defective lysosomal function

**Figure 6:**
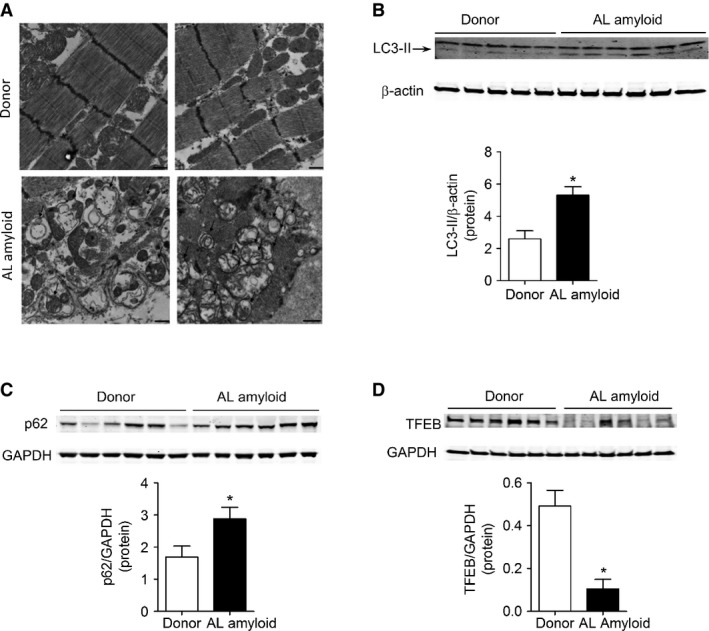
Lysosomal dysfunction and autophagic dysregulation in AL amyloid cardiomyopathy A Transmission electron micrographs of cardiac tissue isolated from donor (top panels) or AL cardiomyopathy patients (bottom panels). Disruption in muscle organization, abnormal mitochondria and double-membrane structures (autophagosomes) are prevalent in heart tissue of AL amyloid cardiomyopathy patients and absent in tissue from healthy donors. Scale bar = 500 nm. B–D Immunoblot analysis for expression of autophagic markers LC3-II (B) and p62 (C) as well as TFEB (D) protein expression in heart tissue isolated from donor or AL cardiomyopathy patients. Immunoblotting revealed increased LC3-II and p62 expression, as well as decreased TFEB protein expression in AL amyloid cardiomyopathy patients compared to healthy donors. *N* = 5 (B), *N* = 6 (C and D). *P = 0.005 (B and C), *P = 0.012 (D). Source data are available online for this figure.

To determine the applicability of our findings to human amyloid cardiomyopathy, we examined markers of autophagy in heart tissue samples obtained from patients with AL amyloid cardiomyopathy. Electron microscopy of human heart tissue revealed dramatic differences in mitochondrial ultrastructure in amyloid cardiomyopathy, with loss of normal spatial distribution between mitochondria and cardiac myofilaments, as well as vacuolization, loss of cristae, swelling and enlargement (Supplementary Fig S4). Significant accumulation of autophagosomes was observed throughout the tissue (Fig 6A) with increased LC3-II and p62 levels (Fig 6B and C) and a decrease in TFEB expression (Fig 6D).

## Discussion

Prior work has detailed an intrinsic cardiotoxic response to human amyloidogenic light chain proteins that underlies the development of AL amyloid cardiomyopathy (Liao *et al*, 2001; Brenner *et al*, 2004; Migrino *et al*, 2010, 2011; Shi *et al*, 2010; Shin *et al*, 2012; Guan *et al*, 2013; Mishra *et al*, 2013). While stress-activated kinases and ROS generation have been identified as downstream components of the cardiomyocyte response to human AL-LC, the fundamental cellular mechanisms underlying the proteotoxicity remain elusive. Here, we find that inhibition of autophagic flux and, specifically, lysosomal dysfunction is central to AL-LC cardiotoxicity and the development of amyloid cardiomyopathy. We further demonstrate that restoration of autophagic flux pharmacologically with rapamycin or genetically through overexpression of TFEB protects against AL-LC cardiotoxicity and may represent a novel therapeutic approach for treatment of amyloid cardiomyopathy.

For our experiments, we utilized human Bence-Jones proteins to study the signaling effects associated with toxic amyloid precursor proteins. We found that light chain solubility is equivalent for AL-LC and Con-LC at the 20 μg/ml concentration used in this study (Supplementary Fig S5A). Additionally, under non-reducing native conditions, we see that the majority of AL-LC and Con-LC proteins migrate to a molecular weight consistent with a dimeric state (Supplementary Fig S5B), while under reducing conditions, these proteins are found at a molecular weight consistent with monomeric state (Supplementary Fig S5C). Further investigation is necessary to determine whether the dimer form of the light chain proteins observed in our study represents a true oligomeric state or merely a state of association. Light chain has two subtypes, lambda and kappa. For our experiments presented in this study, the lambda subtype of AL-LC and kappa subtype of Con-LC proteins were used. Importantly, prior work from our group has found no difference in the cardiotoxic response for amyloidogenic kappa versus lambda light chain proteins (Guan *et al*, 2013; Mishra *et al*, 2013). These observations suggest that both kappa and lambda AL-LC proteins exert a similar cardiotoxic response, whereas neither kappa nor lambda Con-LC proteins resulted in cardiomyocyte toxicity or dysfunction, even at tenfold higher concentrations *in vitro* (100 μg/ml) and *in vivo* (1,000 μg/ml) than routinely used concentrations of AL-LC (Supplementary Fig S6; Mishra *et al*, 2013).

Our previous studies have indicated that ROS generation is a critical factor contributing to AL-LC-induced cellular pathology where phenotypic rescue is seen with antioxidant administration (Brenner *et al*, 2004; Shi *et al*, 2010). Data presented here expand upon our previous work by demonstrating the mitochondrial origin of the increased ROS. Mitochondrial dysfunction was found to be closely associated with AL-LC-induced pathology, resulting from impaired autophagic flux. Dysregulated autophagy has been implicated as key in the pathology of a number of human diseases, ranging from neurodegenerative to cardiovascular diseases, and more recently, protein misfolding diseases including desmin-related cardiomyopathy (Wong & Cuervo, 2010; Bhuiyan *et al*, 2013). Autophagy is a dynamic process that starts with the formation of a double-membrane autophagosome complex that engulfs and then degrades cellular waste products, including organelles such as defective mitochondria. Thus, defects in either formation or clearance of autophagosomes could result in the observed increased levels of LC3-II expression and the formation of double-membrane structures induced by AL-LC. Investigation of each step of the autophagic flux following AL-LC exposure was therefore required to identify the exact point of dysregulation in the autophagy pathway (Mizushima *et al*, 2010). Through use of lysosomal inhibitors, we found that formation of autophagosomes, as measured by LC3-II levels in AL-LC-exposed cardiomyocytes, was similar to control treated cells, indicating that the primary defect in AL-LC exposure is in autophagosome clearance. The autophagic substrate, p62, also known as sequestosome 1 (SQSTM1), is a scaffold protein that binds ubiquitinated protein leading to the formation of autophagosomes for subsequent degradation (Kubli & Gustafsson, 2012). We not only found increased p62 accumulation in our *in vitro* and *in vivo* experimental models in response to AL-LC, but also obser-ved an increase in p62 levels in explanted human hearts from AL cardiomyopathy patients, further supporting a decrease in autophagic clearance as the cause for impaired autophagic flux. It is noteworthy that the increased p62 protein levels most likely resulted from protein accumulation and not increased transcription, as we find that p62 mRNA levels are not increased in AL cardiomyopathy patient hearts compared to control hearts (Supplementary Fig S7).

Recent studies have reported beneficial effects of rapamycin through autophagy activation in a number of experimental disease models (Bove *et al*, 2011; Cortes *et al*, 2012; Cai & Yan, 2013). Restoration of autophagic flux by rapamycin in our system was associated with protection against AL-LC-induced pathology. The beneficial effects of rapamycin were negated with chloroquine, which neutralizes lysosomal pH thereby inhibiting autophagosome clearance, supporting the conclusion that the mechanism of action of rapamycin was primarily through improvement of clearance. Lysosomal function plays a critical role in the clearance of autophagosomes (Eskelinen & Saftig, 2009), and dysfunction or deficiency of the lysosome has been implicated in other amyloid-related diseases, such as Parkinson's disease (Dehay *et al*, 2010). In our disease model, we observed AL-LC-induced loss of lysosomal acidity, with associated downregulation of lysosomal genes and ATPases required for maintenance of pH, as well as decreased TFEB, a critical transcriptional regulator of lysosomes (Settembre *et al*, 2011; Decressac *et al*, 2013; Pastore *et al*, 2013). Furthermore, rapamycin administration resulted in a decrease in autophagosome accumulation and attenuated dopaminergic neuronal cell death, both of which were associated with increased numbers of functional lysosomes (Dehay *et al*, 2010). In our systems, genetic overexpression of TFEB protected against AL-LC cardiotoxicity *in vitro* and *in vivo* and prolonged survival in our zebrafish model. Notably, rapamycin treatment restored TFEB expression to control levels in AL-LC-treated cardiomyocytes, further confirming that its mechanism of action was through targeting lysosomal function.

In summary, the studies presented here show that lysosomal-dependent autophagic dysregulation governs the pathogenesis of AL-LC-induced cellular dysfunction and death. The dysregulation of autophagy leads to the accumulation of depolarized mitochondria, subsequent generation of ROS, and eventual cellular dysfunction and cell death. The therapeutic potential of autophagy-related targets was evident following rescue of AL-LC-induced mortality *in vivo* not only using rapamycin, but also following transient overexpression of TFEB. Our temporal studies suggest that lysosomal insufficiency is among the earliest events that occur in response to AL-LC, and is subsequently followed by dysregulation of autophagy, mitochondrial dysfunction, ROS production, and ultimately overt cellular death and dysfunction. In conjunction with the evidence of profound lysosomal-dependent dysregulation of autophagy in patients with AL amyloid cardiomyopathy, these studies highlight the potential of targeting lysosomal-mediated autophagy as the treatment of the AL amyloid cardiomyopathy patients.

## Materials and Methods

### Human tissues and light chain protein

All procedures related to human light chain protein and heart tissues were reviewed and approved by the Institution Review Board (IRB) at Boston University School of Medicine and Massachusetts General Hospital. Bence-Jones proteins, including amyloidogenic LC isolated from AL amyloid patients (AL-LC) and non-amyloidogenic LC isolated from non-amyloidosis multiple myeloma patients (Con-LC), were obtained from urine purification in collaboration with Boston University Amyloidosis Center (Liao *et al*, 2001; Connors *et al*, 2007). Immunoblotting was used to determine the purity of LC proteins as described previously (Connors *et al*, 2007). Additional information regarding LC protein is listed in Supplementary Table S1. Explanted hearts of patients with AL amyloid cardiomyopathy were collected at the Massachusetts General Hospital. Non-disease control human hearts were purchased from the National Disease Research Interchange. Additional information regarding human samples is listed in Supplementary Table S2.

### Animal care

All animal (rat and zebrafish) procedures were reviewed and approved by the Institutional Animal Care and Use Committee at Harvard Medical School. Rats and zebrafish were housed in Association for Assessment and Accreditation of Laboratory Animal Care (AAALC)-accredited animal care facilities under a 12-h light–dark cycle and were fed with laboratory chow. Adult rats for cardiomyocyte isolation were purchased from Charles River Laboratory (male Wistar rats, 180–220 g, catalog #003). Neonatal rats for cardiomyocyte isolation were purchased from Charles River Laboratory (Wistar rats, p1-p2, catalog #003). Wild-type zebrafish were purchased from Ekkwill Waterlife Resources (Ruskin). Care and breeding of zebrafish were conducted as described previously (Guan *et al*, 2013; Mishra *et al*, 2013).

### Chemicals and reagents

General chemicals and reagents were obtained from Sigma unless otherwise specified. Mito-TEMPO was from Santa Cruz Biotechnology. Low-glucose DMEM with phenol red or without phenol red, TMRE, LysoTracker Red, LysoSensor Blue/Yellow, DCFDA and Laminin were acquired from Invitrogen. Rapamycin was from Cell Signaling Technology. Trypsin and Collagenase were purchased from Willington. Antibodies were obtained from Santa Cruz Biotechnology (TFEB), Cell signaling Technology (total and phosphoryla-ted S6 Kinase, COX4), MBL (LC3), R&D Systems (GAPDH), Sigma (β-actin), Abcam (active caspase 3) and Abnovo (p62). Secondary antibodies for immunohistochemistry (donkey anti-mouse antibody-Alexa Fluor 555 and donkey anti-rabbit antibody-Alexa Fluor 488) were from Invitrogen. Adenovirus purification kit, ATPlite kit and TUNEL kit were purchased from Adenopure, Perkin Elmer and Roche, respectively. Full-length human TFEB-GFP adenovirus was purchased from Vector BioLabs, and adeno-GFP virus was used as a control. MOIs of the two adenovirus were adjusted to infect the adult cardiomyocytes for achieving an equal expression level of GFP as described previously. Experiments were started 24 h following adenovirus infection. Contractile function and intracellular calcium measurements were performed 24 h following AL-LC exposure.

### Cardiomyocyte isolation and culture

As previously described (Jain *et al*, 2003), rat ventricular cardiomyocytes were isolated from adult male Wistar rats using a collagenase-based enzymatic digestion method. Cardiomyocytes were treated with vehicle (ultrapure water), 20 μg/ml of Con-LC or AL-LC at designated time points as described in the Results section. Neonatal rat cardiomyocytes were isolated from 1- to 2-day-old Wistar rats (Charles River Laboratory #003) as previously described (Guan *et al*, 2013).

### Cell contractility measurement and intracellular calcium measurements

Cellular contractile function was measured in cultured adult ventricular cardiomyocytes using video edge detection, and intracellular calcium levels were determined with calcium-sensitive fluorescent dye Fura-2, as described previously (Shi *et al*, 2010). Following treatment or addition of light chain protein, cardiomyocytes were perfused with 1.2 mmol/l Ca^2+^ Tyrode's buffer at 37°C under pacing at 5 Hz. Percentage cellular shortening was calculated as the ratio of the difference between systolic and diastolic cell length over diastolic cell length. Calcium transient amplitude was calculated as the difference in the intracellular calcium levels between systolic and diastolic phases. 4–6 cells were measured per biological replicate, and three biological replicates were performed and grouped for statistical analysis.

### Cell death assays

Cultured cardiomyocytes were washed with PBS and immediat-ely fixed in 4% paraformaldehyde. Cells were permeabilized in pre-chilled methanol at −20°C for 30 min and incubated with TUNEL reaction mixture (Roche) in a moisture chamber for 1 h at 37°C. Slides were washed with PBS for three times and mounted in anti-fade medium containing DAPI (Vectorlabs). For detection of cell death *in vivo*, individual hearts were dissected from zebrafish in Tyrode's solution containing 3% BSA. Hearts were transferred to a microwell plate and fixed in 4% paraformaldehyde for 20 min. Hearts were rinsed in PBS and permeabilized overnight in PBS with 0.1% Tween at 4°C. Hearts were washed three times in PBS, and cell death was detected using TUNEL reaction mixture (Roche) in a moisture chamber for 1 h at 37°C. Hearts were washed with PBS and placed directly into mounting medium containing DAPI. For cardiomyocytes, images were acquired with excitation wavelengths of 405 and 488 nm, and 4–5 pictures were taken from each slide using Axiovision fluorescence microscope (Zeiss). For whole fish hearts, images were taken with excitation wavelengths of 405 and 555 nm, and images were taken using LSM700 confocal microscopy (Zeiss). Percent of apoptotic cell death was calculated as TUNEL-positive nuclei divided by total nuclei. TUNEL-positive nuclei were manually counted, and the total nuclei were counted using ImageJ software (NIH). All of the counting was performed in a blinded fashion. Expression of active caspase 3 for both cell lysates and zebrafish lysate was determined using immunoblotting against active caspase 3.

### RNA isolation and quantitative PCR

To measure gene expression in mRNA level, total RNA was isolated using Trizol (Invitrogen) extraction method. Prior to synthesize cDNA, DNAase treatment was performed subsequently to remove the residual DNA contamination (Turbo DNAase, Ambion). iScript™ cDNA Synthesis Kit (Bio-Rad) was used for first-strand cDNA synthesis. Quantitative PCR was performed using standard curve method using the iCycler PCR (Bio-Rad). The primers are as follows: for rat cardiomyocytes, TFEB forward primer: CTCGAAGTCGGGGAACTAGG, reverse primer: CTGCAGTCGAGGGAAGACAG; GAPDH forward primer: GGTGATGCTGGTGCTGAGTA, reverse primer: TTGCTGACAATCTTGAGGGA; cathepsin D (Cts D) forward primer: GTGGCTTCATGGGGATGGAC, reverse primer: GGAGCAAGTTAGAGTGTGGCA; vacuolar ATPase subunit 1 (VOA1) forward primer: TCTCCACCCATTCAGAGGAC, reverse primer: CCTTCCATGATCAGCAGGAT; vacuolar ATPase subunit 2 (VOA2) forward primer: CAGTTCCGAGACCTCAACCA, reverse primer: GTTTAACAGGTGGTGCGGGA; for fish tissue, TFEB forward primer: GCCACGAGAACGAGATGGAT, reverse primer: GCAGATCCAGACTACCGGGG; and EF1α forward primer: CTGGAGGCCAGCTCAAACATGG, EF1α reverse primer: ACTCGTGGTGCATCTCAACAGACT.

### Immunohistochemistry

Following treatment, adult cardiomyocytes were washed twice with 1× PBS and then fixed/permeabilized with acetone/methanol (1:2) solution at −20°C for 20 min. Incubation with 3% BSA solution for 1 h at room temperature was performed to minimize non-specific binding. Cells were then incubated with two primary antibodies (anti-p62 [1:100] and COX4 [1:1,000]) at 4°C for 18 h followed by subsequent incubation with secondary antibodies (donkey anti-mouse Alexa Fluor 555 [1:300] and donkey anti-rabbit Alexa Fluor 488 [1:300]) for 1 h at 37°C to detect p62 and COX4, respectively. After final washing with PBS, the slides were mounted with VECTASHIELD® mounting media (Vector Lab). Zeiss LSM700 fluorescence confocal microscope was used to visualize p62 (Ex/Em: 550/600 nm) and COX4 (Ex/Em: 488/525 nm) with 63× lens. DAPI was used to stain for the nuclei.

### Immunoblot

For rat cardiomyocytes, protein was extracted using cell lysis buffer (Cell Signaling) with 1 mM PMSF (Sigma) and then subjected to sonication. The protein concentration was determined by Dc protein assay (Bio-Rad). For zebrafish, 15 embryos were suspended directly in 50-μl SDS loading buffer and homogenized using a tissue homogenizer (TissueLyser II, Qiagen). Following homogenization, the samples were centrifuged and total protein homogenates were obtained. 30 μg of total protein or 18 μl of fish protein lysate was loaded onto Criterion XT bis-tris precast gels (4–12%) (Invitrogen) or PAGEr Gold precast gels (4–20%) (Lonza) for electrophoresis. Protein was electrotransferred to a PVDF membrane (Millipore) at 30 volts for 16–18 h at 4°C. After blocking in 5% BSA in PBS, proteins of interest were detected by incubation with appropriate primary antibodies overnight at 4°C. After washing, blots were incubated with corresponding secondary antibodies. Odyssey infrared scanner (Li-Cor) was used to determine the infrared fluorescent signal, and GAPDH was used as a reference gene for normalization.

### Mitochondrial membrane potential and ATP measurement

Following Con-LC, AL-LC (20 μg/ml) or vehicle administration for designated number of hours, cultured cardiomyocytes were incubated with cell permeable, mitochondrial membrane potential-sensitive fluorophore TMRE (Invitrogen) at the concentration of 10 nM for 30 min. Cardiomyocytes were then washed with warm PBS 2 times. TMRE fluorescence was acquired with excitation wavelengths of 555 nm, and 4–5 pictures were taken from each dish using LSM700 confocal microscopy (Zeiss). Mean fluorescence intensity of individual cardiomyocytes was determined per picture with ImageJ software (NIH). Cellular ATP level was determined with ATPlite kit according to the manufacture's manual. Briefly, cardiomyocytes were cultured on a 12-well plate. Following 24-h treatment of LCs, warm PBS was used to wash cells gently. 75 μl of PBS was added to each well, followed by 75 μl of cell lysis buffer. Plate was subjected to 5-min shaking at 700 r.p.m. to break up the plasma membrane. 150 μl of ATP luminescent reaction buffer with substrate was added into each well, and the luminescence signal was measured with a SpectraMax M5 Microplate Reader (Molecular Device).

### ROS measurement

Following Con-LC or AL-LC (20 μg/ml) or vehicle administration for designated number of hours, cultured cardiomyocytes were incubated with cell permeable, redox-sensitive fluorophore DCFDA (Invitrogen) at the concentration of 20 μM for 30 min. Cardiomyocytes were then washed with warm PBS 2 times. Cell images were acquired using LSM700 confocal microscopy (excitation wavelength at 488 nm) and analyzed with SigmaScan Pro. For determination of mitochondrial-derived ROS, cardiomyocytes were pre-treated with Mito-TEMPO (Santa Cruz Biotech), a mitochondrial-specific ROS scavenger, at a concentration of 100 nM for 45 min prior to experimental manipulation.

### Autophagic flux measurement

For autophagosome clearance, adult rat cardiomyocytes were treated with either Con-LC or AL-LC (20 μg/ml) or vehicle for 24 h. Autophagy-specific substrate p62 was measured with immunoblotting. For autophagosome generation rate, adult rat cardiomyocytes were treated with either AL-LC (20 μg/ml) or vehicle for 48 h in the presence or absence of lysosomal inhibitors (E64d and Pepstatin A at the concentration of 5 μg/ml) (Hamacher-Brady *et al*, 2006). LC3-II levels were then measured with immunoblotting.

### GFP-LC3 cleavage assay

Neonatal cardiomyocytes were infected with GFP-LC3 adenovirus. Twenty-four hours following adenoviral infection, neonatal cardiomyocytes were exposed to either vehicle, Con-LC or AL-LC for 2, 6 or 12 h. Following 1× PBS wash, cells were manually harvested using a cell lifter. Protein homogenate from harvested lysed cells are then subjected to immunoblotting for GFP. GFP antibody was used to detect the presence of GFP-LC3 fusion protein. Degradation of the fusion protein is reduced under conditions when autophagic flux is inhibited (Ni *et al*, 2011).

### LysoTracker and LysoSensor staining

Following designated hour treatment with vehicle, Con-LC and AL-LC (20 μg/ml), cultured cardiomyocytes were incubated with cell permeable, lysosomal-specific probe LysoTracker (Invitrogen) at a concentration of 100 nM for 30 min. Cardiomyocytes were washed twice with warm PBS. Cell images were acquired using LSM700 confocal microscopy (excitation wavelength at 555 nm) and analyzed with ImageJ software. For determination of lysosomal pH, cardiomyocytes were incubated with LysoSensor Blue/Yellow (Invitrogen) at a concentration of 1 μM for 3 min prior to measurement using LSM710 two-photon confocal microscopy (excitation wavelength was 720 nm, and emission wavelengths were collected from 400 to 461 nm for the blue emission and 510 to 630 nm for the yellow emission). Images were analyzed using imageJ software. Green fluorescence signal (blue emission) represents basic conditions, and red fluorescence signal (yellow emission) represents acidic conditions. Pseudocoloring (the ratio of green/red) was done to represent lysosomal pH.

### Electron microscopy

1-mm^3^ cubes of human heart samples from either non-failing control or AL amyloid cardiomyopathy patients, or whole zebrafish embryos that had been injected with Con-LC or AL-LC, were fixed with 2.5% glutaraldehyde overnight at 4° and then embedded in epoxy resins. Ultrathin sections (80 nm) were stained with uranyl acetate/lead citrate and then examined under the Tecnai G² electron microscope (FEI Inc) in Harvard Medical School EM core facility.

### Assessment of cardiac function in zebrafish

Either Con-LC or AL-LC (100 μg/ml) was introduced into zebrafish circulation via venous injection as previously described. Following randomization, fish were treated with vehicle or rapamycin [10 nM] (Tobin & Beales, 2008). 5dpf zebrafish were embedded in 4% low-melting agarose (Invitrogen) made with E3 water. E3 water was added to a level of 2 mm above the agarose. Color Doppler echocardiography was performed using MS700 probe (Vevo2100, VisualSonics) to determine the peak aortic flow velocity at 50 MHz. The color Doppler gate was placed on the dorsal edge of ventricle. The maximal flow velocity of each fish was acquired in a blinded fashion. Per each animal, the acquisition part starting at embedding was kept within 3 min to ensure fish health during time of measurement. 6–8 fish were examined for each group.

### Transient TFEB overexpression zebrafish model

A pair of primers was designed to amplify fish TFEB mRNA from whole fish mRNA samples: upstream primer: ATTTAGGTGACACTATAGAAATGTCGTCACGCATCGGCCT; downstream primer: CCGCTCGAGTCACTGTATATC. mMESSAGE mMACHINE Kit (Invitrogen) was used to synthesize zebrafish TFEB mRNA. Reverse TFEB mRNA was synthesized for control. The quality/quantity of mRNA was determined by Nanodrop spectrometer (Thermo Scientific). 20 pg of mRNA was injected into zebrafish embryos at single cell stage. AL-LC was introduced into fish circulation via venous injection 2 days post-fertilization. Cardiac cell death was determined at 4dpf. Fish survival was monitored until day 7 post-fertilization.

### Statistical analysis

All data are shown as mean ± standard error. Statistical differences between mean values for two groups were evaluated by Student's *t*-test using GraphPad Prism software and confirmed using Microsoft Excel. Individual *P*-values are denoted within the figure legends. *P* < 0.05 was considered as significant.
