# Supplementary material for: Lysosomal dysfunction and impaired autophagy underlie the pathogenesis of amyloidogenic light chain-mediated cardiotoxicity
Source: EMBO Mol Med. 2014 Oct 15;6(11):1493–507. doi: 10.15252/emmm.201404190 (PMC4237473; doi:10.15252/emmm.201404190)
Supplement: Supplementary file 1 [file emmm0006-1493-sd1.pdf]

EMBO Molecular Medicine

Lysosomal dysfunction and impaired autophagy underlie the pathogenesis of amyloidogenic light chain mediated cardiotoxicity

Jian Guan, Shikha Mishra, Yiling Qiu, Jianru Shi, Kyle Trudeau, Guy Las, Marc Liesa, Orian S. Shirihai, Lawreen H. Connors, David C. Seldin, Rodney H. Falk, Calum A. MacRae and Ronglih Liao

Corresponding author: Ronglih Liao, Brigham and Women's Hospital, Harvard Medical School

Review timeline:

Submission date:	21 April 2014
Editorial Decision:	20 May 2014
Revision received:	08 August 2014
Editorial Decision:	10 September 2014
Revision received:	19 September 2014
Accepted:	19 September 2014

Transaction Report:

(Note: With the exception of the correction of typographical or spelling errors that could be a source of ambiguity, letters and reports are not edited. The original formatting of letters and referee reports may not be reflected in this compilation.)

Editor: Roberto Buccione

1st Editorial Decision 20 May 2014

Thank you for the submission of your manuscript to EMBO Molecular Medicine. We have now heard back from the three Reviewers whom we asked to evaluate your manuscript.

You will see that the three Reviewers, while mostly acknowledging the interest of your work, express several, partially overlapping concerns that prevent us from considering publication at this time. My overall impression is that all Reviewers are dissatisfied (to varying degrees) with various aspects including insufficient experimental support and lack of convincing translational impact. I would like to just mention a few specific points without dwelling into much detail, as the evaluations are detailed and self-explanatory.

Reviewer 1 is quite positive but would like to see additional details on the biophysical properties of the light chains and suggests additional controls in this respect. S/he also notes the lack of a dose-response study (see also below comments from Reviewer 2). Finally, this Reviewer requires more patient information to boost the clinical relevance of the manuscript.

Reviewer 2 is especially concerned about the lack of and dose- (as Reviewer 1) and especially time-dependence of the reported effects. I agree that this is a critical experimental shortcoming that needs to be fully addressed to afford clear relevance to the findings.

Reviewer 3 is more negative and specifically questions the translational relevance of the findings as they stand (similarly to Reviewer 1), especially due to the lack of a clear understanding of the relationships with the other mechanisms of toxicity.

For the above reasons, while publication of the paper cannot be considered at this stage, we would be prepared to examine a substantially revised submission, with the understanding that the Reviewers' concerns must be fully addressed with additional experimental data where appropriate and that acceptance of the manuscript will entail a second round of review.

***** Reviewer's comments *****

Referee #1 (Comments on Novelty/Model System):

the model is fine, I think that more details are required

Referee #1 (Remarks):

The paper of Guan et al. "Impaired autophagy and lysosomal dysfunction underlie the pathogenesis of amyloidogenic light chain mediated cardiotoxicity" reports interesting new data related to the mechanism of cardiotoxicity of amyloidogenic light chains.

The data are robust, well discussed and technology is at the state of the art. However I think that the authors should clarify a few points.

1. Which are the biochemical characteristics of the amyloidogenic and non-amyloidogenic light chains (i.e. solubility, state of association -monomeric-dimeric -oligomeric and in which proportion)? From the table I have the impression that the control is a k light chain. An equivalent number of non-cardiotoxic lambda light chains are necessary.

2. There're no data regarding a dose effect response. I'm wondering if the control light chains can become toxic at higher concentration

3. The results obtained on the ex vivo tissues are consistent with the data derived from the experimental model. The authors should report the data on the concentration of free light chains in these patients at the time of heart explantation and a few clinical data. The patients were treated with any type of chemotherapy? Does the control tissue were derived from patients treated with similar treatment. Data of each single patient's tissue should be reported and I'm wondering if a correlation exists between the free light chain concentration and parameters of autophagy impairment

Referee #2 (Remarks):

This paper's topic is the pathogenesis amyloid-light chain cardiomyopathy and suggests that activation of autophagy might be an therapeutic option. The latter is an interesting and novel suggestion for stalling AL-LC toxicity. The pathogenesis part explores known pathways involved in involved in cardiac homeostasis and disease in the context of AL-LC toxicity. The experimental approaches, the animal model, and patient samples are well chosen and executed, with one major caveat:
Development of AL-LC toxicity (as well as pathogenesis of any disease) is an temporal order of molecular and cellular events. The current manuscript does not contain time-resolved analyses of the processes studied. Rather, it is hard to extract any information on the time points studied - apparently most assays have been done at 24h. Thus it is not possible to say which process comes first and "what is causing what". ROS?, Mitocondrial damage? Defective autophagy? Defective lysosomes? Thus the present manuscript does not live up to its title. Based on the data presented it is impossible to place autophagy and lysosomal dysfunction properly in AL-LC pathogenesis. Presentation of time resolved data of the key cell culture experiments of this study is necessary to establish a valid sequence of events upon AL-LC exposure.

Referee #3 (Comments on Novelty/Model System):

in human hearts, additional mechanisms of toxicity and cell death might operate, including those related with intermediary amyloidogenic species. Therefore extrapolation of the "in vitro" to the "in vivo" data is limited.

Referee #3 (Remarks):

The work by Guan et al. addresses autophagic flux dysregulation by amyloidogenic LC associated with amyloid cardiopathy. By a series of studies, several parameters with autophagy were assessed at the cellular level and in zebra fish comparatively to control non-amyloidotgenic LC; furthermore, restoration of the toxic phenotype was achieved by ripamycin. The experiments were well designed and elucidative of autophagic flux dysregulation in the used systems.

Autophagic flux deregulation and rescue by ripamycin is common in degenerative disorders. Generally missing in the present manuscript is co-relation of mechanisms of toxicity in the LC system. For instance, with mitochondrial toxicity, calcium dysregulation previously published by the authors ? traffic of the LC to lysosomes/autophagosomes and thereafter ?; in human hearts, additional mechanisms of toxicity and cell death might operate, including those related with intermediary amyloidogenic species.

Other points:

- the panels of figure 3, specially westerns blots are not of high quality and should be improved.
- Experimental details with adenovirus work are not described in the Materials and Methods section.

1st Revision - authors' response 08 August 2014

Response to Referee 1

The authors thank the referee for the critical comments. We have revised our manuscript with more detail information as recommended, as described below.

"The model is fine, I think that more details are required"

Response - as requested, we have included additional details of the model and human light chain proteins used, as described below.

"Which are the biochemical characteristics of the amyloidogenic and non-amyloidogenic light chains (i.e. solubility, state of association -monomeric-dimeric -oligomeric and in which proportion)? From the table I have the impression that the control is a k light chain. An equivalent number of non-cardiotoxic lambda light chains are necessary."

Response - The referee raises an important question. We have now performed additional biochemical characterization of the amyloidogenic and non-amyloidogenic light chain (LC) proteins used in the study. Using a well-described turbidity assay, we found that light chain solubility is equivalent for AL-LC and control (Con)-LC at the 20 mg/ml concentration used in this study (Figure Expanded 5A). Using non-reducing native gel electrophoresis, we have found that the majority of both AL-LC and Con-LC protein exists in the dimeric state (Figure Expanded 1B and C). We have now incorporated these new data into the results section (pages 10, para 3, lines 2-5, Figure E5B-C)

We agree with the referee that under ideal circumstances, we would have included an equivalent number of non-cardiotoxic lambda light chain proteins for study. Light chain proteins were obtained from collected urine specimens from patients presenting to the BU Amyloidosis Center, and are extremely limited in type and quantity. No additional control lambda light chain protein samples were available at the time of this study. Importantly, though, prior work from our group has found no difference in the cardiotoxic response for kappa versus lambda light chain proteins (Mishra S, et al. Am J Physiol Heart Circ Physiol. 2013 Jul 1;305(1):H95-103.; Guan J, et al. Basic Res Cardiol. 2013 Sep;108(5):378). This observation has been confirmed by independent reports (Migrino RQ, et

al. Int J Cardiol. 2010 Nov 5;145(1):67-68; Sikkink LA et al. Cell Death Dis. Nov 2010; 1(11): e98). These observations suggest that both kappa and lambda light chain proteins exert a similar cardiotoxic response when isolated from cardiac amyloidosis patients. We have amended the manuscript to make clear mention of this point (page 11, para 1, lines 1-4).

"There're no data regarding a dose effect response. I'm wondering if the control light chains can become toxic at higher concentration."

Response – we have previously shown using this same zebrafish model that control light chain proteins do not exhibit a cardiotoxic response, even at 10X the concentration of amyloidogenic light chain proteins (Mishra S, et al. Am J Physiol Heart Circ Physiol. 2013 Jul 1;305(1):H95-103]. This point is now stated in the revised manuscript (page 11, para 1, lines 6-8). In addition, we performed experiments using a 10X higher concentration of Con-LC in isolated cardiomyocytes and found no difference in contractile function and calcium homeostasis compared to control conditions (Figure E6 page 11, para 1, lines 6-8).

"The results obtained on the ex vivo tissues are consistent with the data derived from the experimental model. The authors should report the data on the concentration of free light chains in these patients at the time of heart explanation and a few clinical data. The patients were treated with any type of chemotherapy? Does the control tissue were derived from patients treated with similar treatment. Data of each single patient's tissue should be reported and I'm wondering if a correlation exists between the free light chain concentration and parameters of autophagy impairment."

Response – Unfortunately the free light chain concentrations were not available for these specific patients at the time of cardiac explantation. Importantly, though, circulating light chain concentrations in our zebrafish model are comparable to those typically observed in patients with AL amyloidosis (Kumar S, et al. Blood. 2010 Dec 9;116(24):5126-5129). Furthermore, the human cardiac tissues were obtained under strict IRB protocol as de-identified samples. Given that cardiac amyloidosis is a relatively rare disease, it was felt by the IRB that disclosing any clinical or demographic data for patients could compromise patient anonymity, thus, clinical data and treatment history are not available. Control tissues were obtained from non-suitable cardiac donors, as is standard practice. While it would be interesting to determine if any association exists between concentrations of circulating free light chain and parameters of autophagy impairment in humans, a statistically significant analysis would likely require many more patient samples than were available for our studies, particularly given disease and clinical heterogeneity. As indicated by the referee, evidence for impaired autophagy in human ex vivo tissue from patients with amyloid cardiomyopathy was highly consistent with the cardiotoxic response observed in vivo in our experimental zebrafish model, suggesting that this cardiotoxic response is indeed conserved among model systems.

Response to Referee #2

We thank the Referee for their time and helpful comments. Our response is as detailed below:

"Development of AL-LC toxicity (as well as pathogenesis of any disease) is an temporal order of molecular and cellular events. The current manuscript does not contain time-resolved analyses of the processes studied. Rather, it is hard to extract any information on the time points studied - apparently most assays have been done at 24h. Thus it is not possible to say which process comes first and 'what is causing what'. ROS? Mitochondrial damage? Defective autophagy? Defective lysosomes? Thus the present manuscript does not live up to its title. Based on the data presented it is impossible to place autophagy and lysosomal dysfunction properly in AL-LC pathogenesis. Presentation of time resolved data of the key cell culture experiments of this study is necessary to establish a valid sequence of events upon AL-LC exposure."

Response – The Referee has raised an important question. We have now performed additional time course experiments to determine the temporal relationship among the critical components of the AL-LC cardiotoxic response. We find that upon AL-LC exposure, decreased lysosomal function is observed very early (within 3 hours). Lysosomal dysfunction is followed by dysregulation of autophagy at 6 hours post AL-LC exposure. Decreased mitochondrial membrane potential also occurred at 12 hours; increased ROS production was most pronounced at 24 hours. Lastly, overt cellular dysfunction, marked by depressed cardiomyocyte contractility, and impaired calcium handling is seen at 24 hours post AL-LC. Cellular death is observed at 48 hours. These new data suggest that lysosomal dysfunction is among the earliest events that occur in response to AL-LC, and this subsequently initiates dysregulation of autophagy, mitochondrial dysfunction, ROS production, and ultimately overt cellular death and dysfunction. These data now place lysosomal and autophagy dysfunction as early events in the pathogenesis of AL-LC cardiotoxicity. These new data and a flowchart detailing the temporal sequence of cellular events have been included in the revised manuscript (Fig 5; page 9, para 2, lines 1-11; page 13, para 2, lines 6-9.

Referee #3 (Comments on Novelty/Model System):

We thank the Referee for the helpful comments and review. Our response to the Referee's comments is detailed below:

"In human hearts, additional mechanisms of toxicity and cell death might operate, including those related with intermediary amyloidogenic species. Therefore extrapolation of the "in vitro" to the "in vivo" data is limited."

Response – We agree with the referee that in humans additional mechanisms may contribute to the AL amyloid cardiomyopathy. Importantly, our in vitro cellular and in vivo zebrafish data have revealed a new molecular insight into the underlying pathogenesis of AL light chain cardiotoxicity and we have further confirmed that these mechanisms, namely impaired lysosomal clearance and dysregulation of autophagy, are also present in cardiac tissue from patients with AL amyloid cardiomyopathy, suggesting that those mechanisms identified in our experimental systems are relevant in humans. We hope that our observations will serve as the foundation for such future laboratory and clinical studies to validate our hypothesis.

"Autophagic flux deregulation and rescue by rapamycin is common in degenerative disorders. Generally missing in the present manuscript is co-relation of mechanisms of toxicity in the LC system. For instance, with mitochondrial toxicity, calcium dysregulation previously published by the authors ? traffic of the LC to lysosomes/autophagosomes and thereafter ?; in human hearts, additional mechanisms of toxicity and cell death might operate, including those related with intermediary amyloidogenic species."

Response – we agree with the Referee that it is of importance to identify the relationship between the mechanisms identified in this paper, including lysosomal and autophagic dysfunction, and previously identified mechanisms of toxicity, including mitochondrial dysfunction (Fig 2B-C) and impaired calcium homeostasis (Fig 2E and Fig 4G). As detailed above in our response to Referee #2, we have now performed additional time course experiments to determine the temporal relationship among the critical components of the AL-LC cardiotoxic response (Fig 5). We find that upon AL-LC exposure, lysosomal dysfunction is observed very early (within 3 hours). Lysosomal dysfunction is followed by dysregulation of autophagy at 6 hours post AL-LC exposure. Decreased mitochondrial membrane potential and associated increased ROS production also occurred at 6 hours and were most pronounced at 12 hours following AL-LC. Lastly, overt cellular dysfunction, marked by depressed cardiomyocyte contractility, impaired calcium handling, and cellular death, occurs at 48 hours post AL-LC. These new data suggest that lysosomal dysfunction is among the earliest events that occur in response to AL-LC, and this subsequently initiates dysregulation of autophagy, mitochondrial dysfunction, ROS production, and ultimately overt cellular death and dysfunction. As suggested by Referee 2 and 3, these data place lysosomal and autophagy dysfunction as temporally early events in the pathogenesis of AL-LC cardiotoxicity. These new data

and a flowchart detailing the temporal sequence of cellular events have been included in the revised manuscript (Fig 5; page 9, para 2, lines 1-11; page 13, para 2, lines 6-9).

The trafficking of light chain to other cellular compartments is an important point, and interesting question with regards to the signalling mechanisms involving precursor proteins. While this issue is outside of the scope of the current studies, this question is something that our lab is actively investigating right now, and studies are currently on-going.

As presented in the current manuscript, we find evidence for lysosomal dysfunction and dysregulation of autophagy in cardiac tissue obtained from patients with AL amyloid cardiomyopathy relative to control patients. While we agree that additional mechanisms of toxicity and cell death may operate in humans with complex disease, to definitively determine the role of lysosomal and autophagic dysfunction in human disease would require a randomized controlled trial of rapamycin in patients. Such a trial is of great importance and we hope that the observations detailed in our paper will serve as the foundation for such future clinical studies in AL amyloid cardiomyopathy.

"the panels of figure 3, specially westerns blots are not of high quality and should be improved."

Response – new Western blot figures have been included in the revised manuscript of higher quality (Fig 3A, 3B and 3D).

"Experimental details with adenovirus work are not described in the Materials and Methods section."

Response – We apologize for this oversight. Methods describing the adenovirus work have now been included in the revised manuscript (page 15, para 2, lines 11-16).

2nd Editorial Decision 10 September 2014

Thank you for the submission of your revised manuscript to EMBO Molecular Medicine. We have now received the enclosed reports from the referees that were asked to re-assess it. As you will see, while all three Reviewers are now supportive, Reviewer 1 would like more convincing evidence on the state of dimeric Bence Jones. I will not be asking you to perform further experimentation at this stage (although, if available, I would strongly advise including it) but I suggest you to be more cautious in the manuscript text and revise accordingly. I will therefore be able to accept your manuscript pending the above.

Please submit your revised manuscript within two weeks. I look forward to seeing a revised form of your manuscript as soon as possible.

I look forward to reading a new revised version of your manuscript as soon as possible.

***** Reviewer's comments *****

Referee #1 (Remarks):

I think that the authors have done all the reasonable efforts for answering the questions I raised. I'm still convinced that more control with non amyloidogenic light chains would be necessary as well as more exhaustive clinical information on the patients from which biopsies were taken.
I have only a technical request regarding the determination of dimeric
Bence Jones. In order to prove that the dimers are linked through the C-terminal cya an SDS PAGE in non reducing conditions should be carried out. Regarding the definition of oligomeric state fro the

dimers I think it is not appropriate. It is just the state of association of a native self limiting dimer with a proper quaternary structure.

Referee #2 (Remarks):

This manuscript has been greatly improved upon revision. Specifically, my comments concerning the temporal order of events have been sufficiently addressed.
I have one minor comment: the title of the paper should also reflect the order of events. I believe it would be more appropriate to state: "Lysosomal dysfunction and impaired autophagy underlie..."

Referee #3 (Remarks):

The information on temporal analyses of cellular events occurring upon incubation was very valuable showing autophagy as the first event in cell dysfunction. The traffic of the amyloidogenic LC to lysosomes will be important as but understand needs additional work to be included elsewhere.

2nd Revision - authors' response 19 September 2014

The authors thank the referees for their critical comments. We have revised our manuscript with more detailed information as recommended, as described below.

Response to Referee 1

"I think that the authors have done all the reasonable efforts for answering the questions I raised. I'm still convinced that more control with non amyloidogenic light chains would be necessary as well as more exhaustive clinical information on the patients from which biopsies were taken. I have only a technical request regarding the determination of dimeric Bence Jones. In order to prove that the dimers are linked through the C-terminal cya an SDS PAGE in non reducing conditions should be carried out. Regarding the definition of oligomeric state fro the dimers I think it is not appropriate. It is just the state of association of a native self limiting dimer with a proper quaternary structure. "

Response – The authors fully agree with the reviewer's comment and we have amended the manuscript to include "Additionally, under non-reducing native conditions, we see that the majority of AL-LC and Con-LC proteins migrate to a molecular weight consistent with a dimeric state (Fig E5B), while under reducing conditions, these proteins are found at a molecular weight consistent with monomeric state (Fig E5C). Further investigation is necessary to determine if the dimer form of the light chain proteins observed in our study represents a true oligomeric state or merely a state of association." (page 10, para3, line 4-5 and page 11, para 1, line 1-4).

Response to Referee #2

"This manuscript has been greatly improved upon revision. Specifically, my comments concerning the temporal order of events have been sufficiently addressed. I have one minor comment: the title of the paper should also reflect the order of events. I believe it would be more appropriate to state: "Lysosomal dysfunction and impaired autophagy underlie..."

Response – Thank you for an excellent suggestion. As suggested, we have changed the title accordingly. Now, the new title reads "Lysosomal dysfunction and impaired autophagy underlie the pathogenesis of amyloidogenic light chain mediated cardiotoxicity"

Response to Referee #3

"The information on temporal analyses of cellular events occurring upon incubation was very valuable showing autophagy as the first event in cell dysfunction. The traffic of the amyloidogenic LC to lysosomes will be important as but understand needs additional work to be included elsewhere.."

Response – Thank you.
