# Supplementary material for: Lysosomal dysfunction and impaired autophagy underlie the pathogenesis of amyloidogenic light chain-mediated cardiotoxicity
Source: EMBO Mol Med. 2014 Oct 15;6(11):1493–507. doi: 10.15252/emmm.201404190 (PMC4237473; doi:10.15252/emmm.201404190)

Source data for Figure 2A. The red box indicates the cropped image used in the manuscript figure. Left panel: WB using anti-p62 was imaged at 800 nm. Right panel: the same WB using anti-GAPDH was imaged at 700 nm as a loading control.

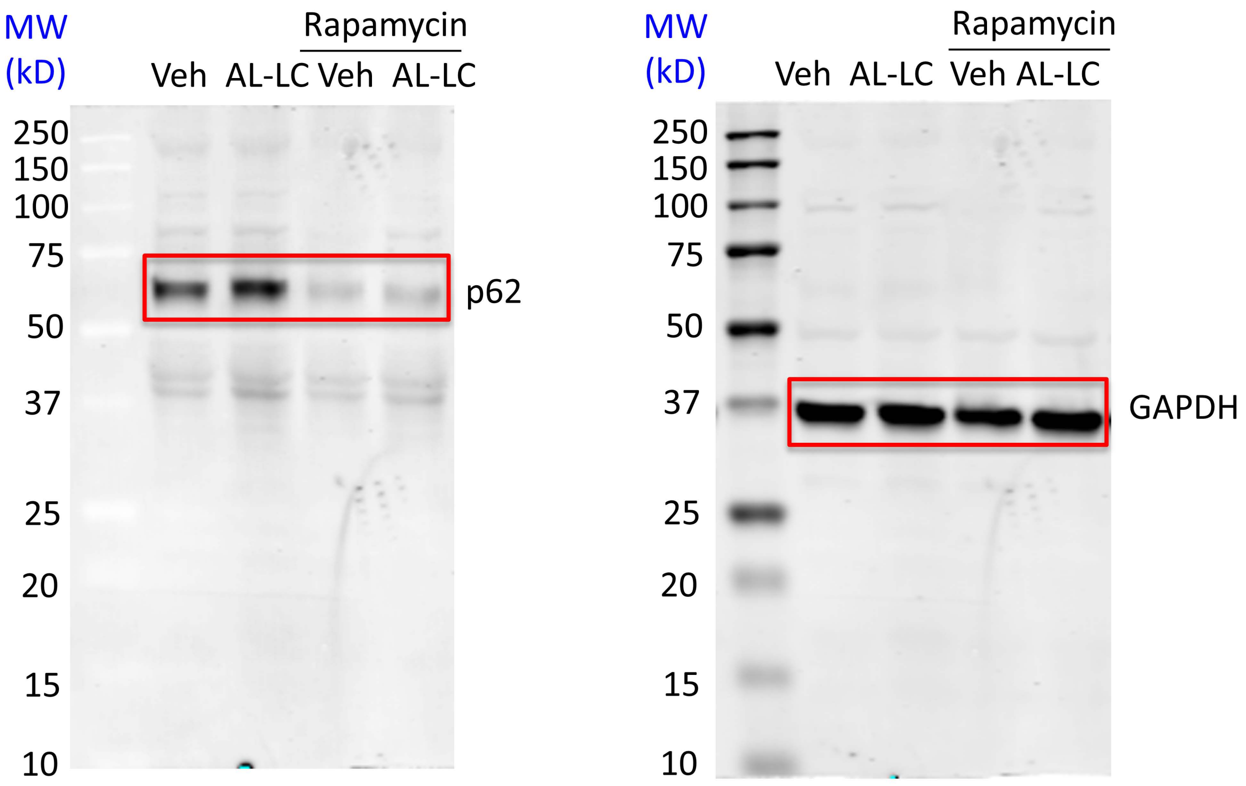

Source data for Figure 2G. Left panel: WB using anti-p62 was imaged at 800 nm. Right panel: the same WB using anti-GAPDH was imaged at 700 nm as a loading control.

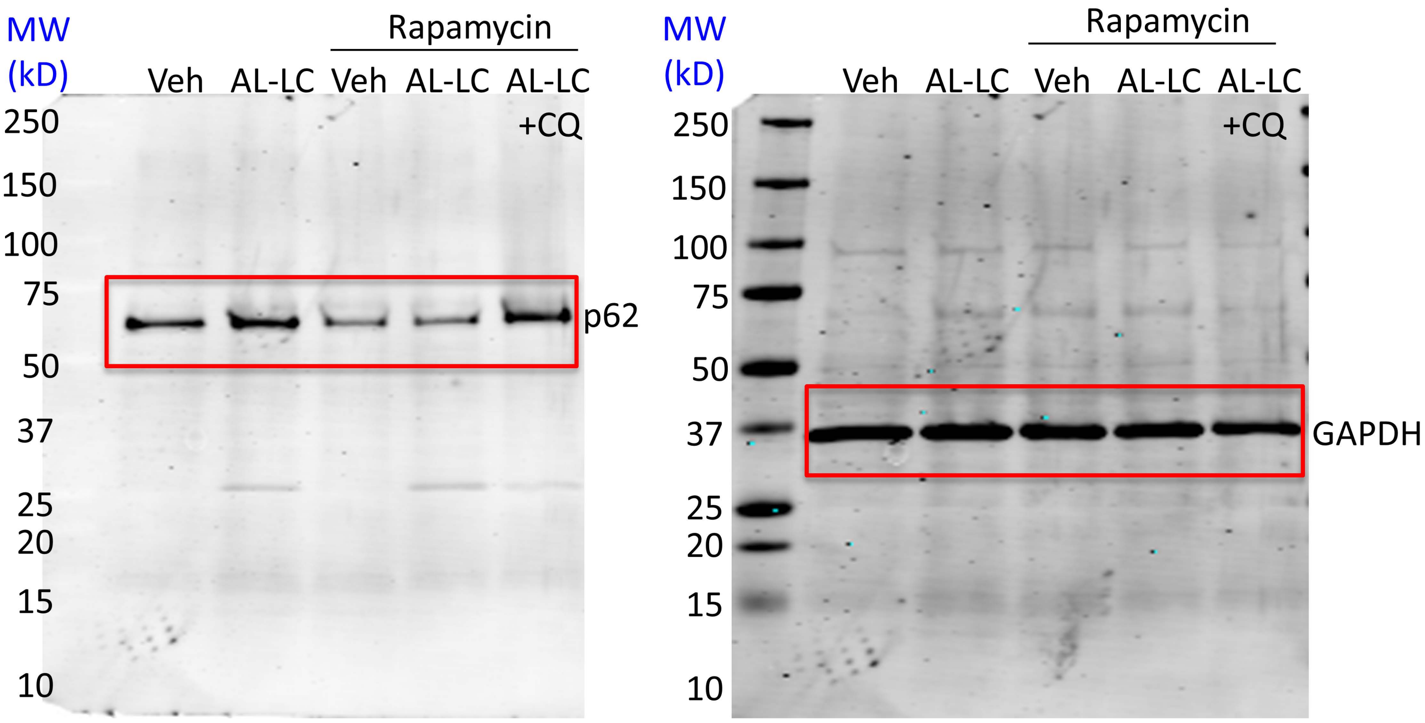

Source data for Figure 2H. Left panel: WB using anti-active caspase 3 was imaged at 700 nm. Right panel: membrane was stripped and reblotted using anti-GAPDH and imaged at 700 nm as a loading control.

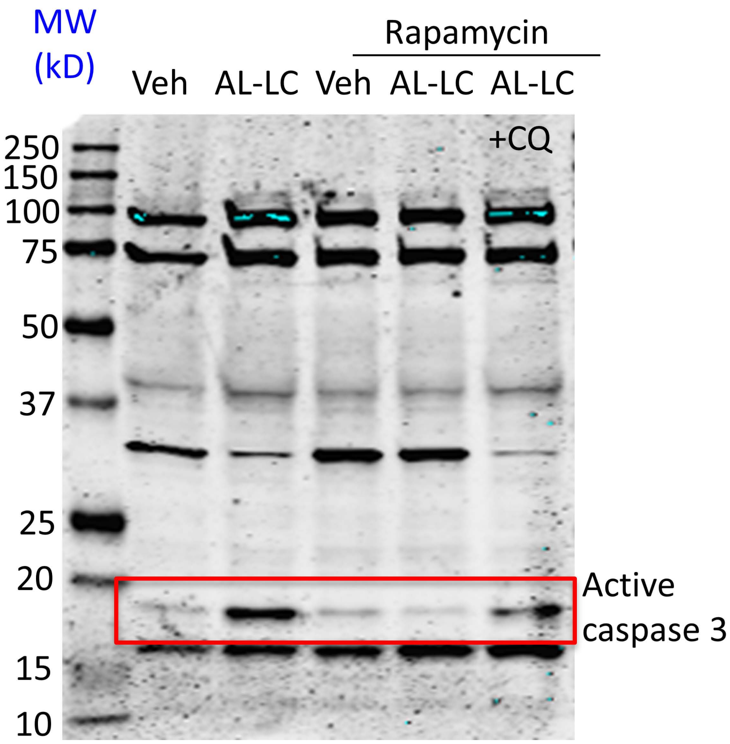

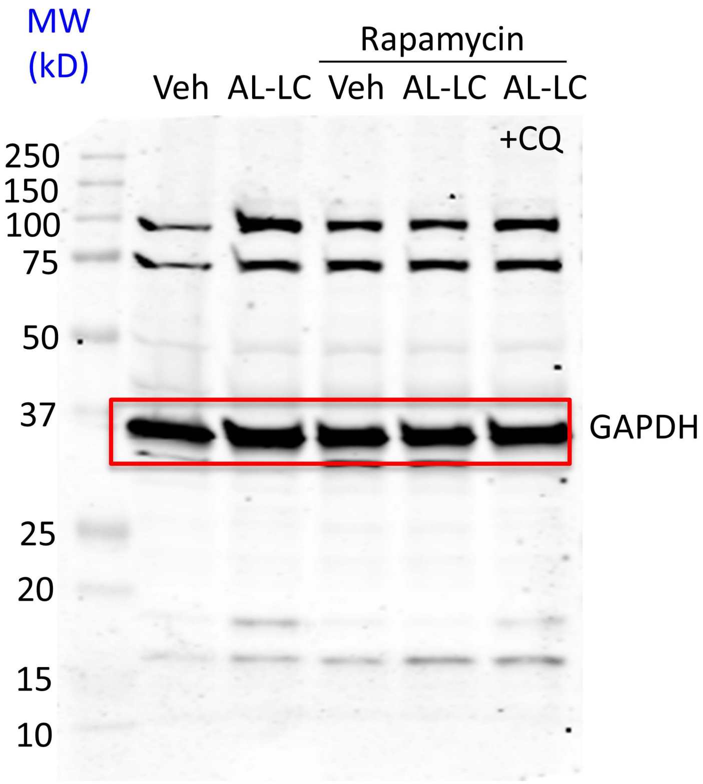

Supplement: Supplementary file 3 [file emmm0006-1493-sd3.pdf]
