# Supplementary material for: Lysosomal dysfunction and impaired autophagy underlie the pathogenesis of amyloidogenic light chain-mediated cardiotoxicity
Source: EMBO Mol Med. 2014 Oct 15;6(11):1493–507. doi: 10.15252/emmm.201404190 (PMC4237473; doi:10.15252/emmm.201404190)

Source data for Figure 3A. The red box indicates the cropped image used in the manuscript figure. Left panel: WB using anti-LC3 was imaged at 700 nm. Right panel: membrane was stripped and reblotted using anti-GAPDH and imaged at 700 nm as a loading control.

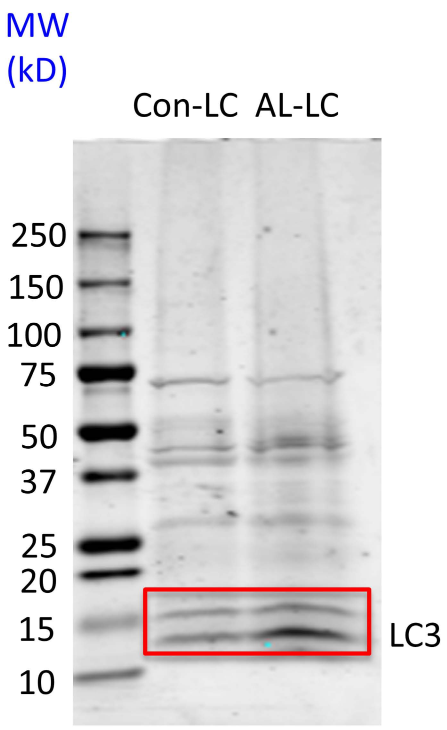

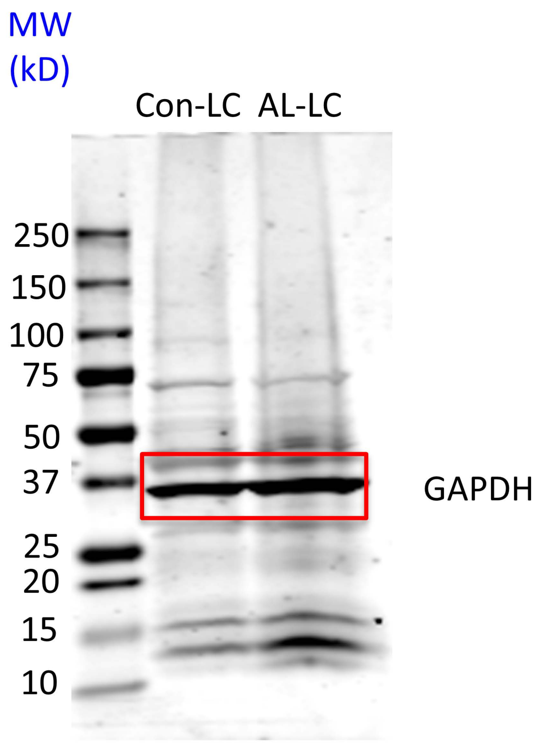

Source data for Figure 3B. Left panel, WB using anti-p62 was imaged at 800 nm.   Right panel: the same WB using anti-GAPDH was imaged at 700 nm as a loading control.

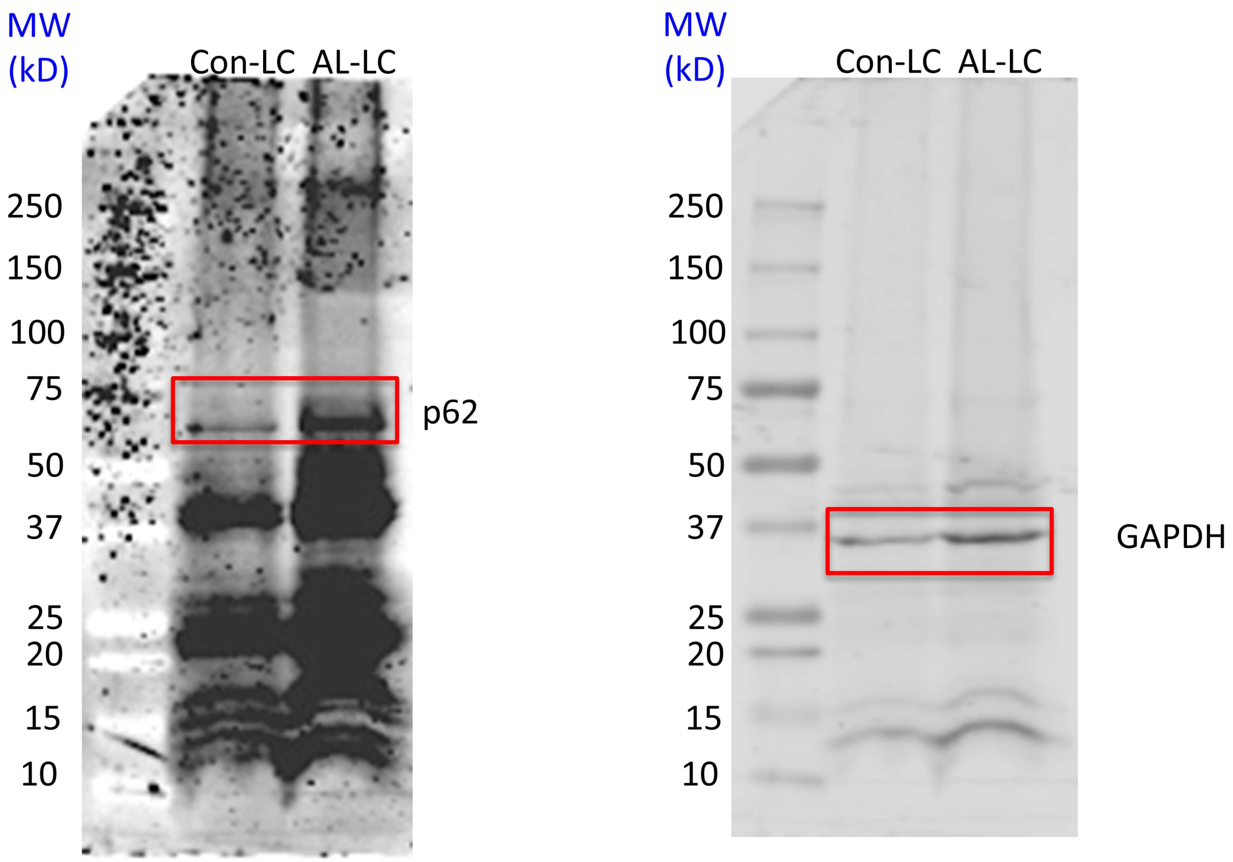

Source data for Figure 3D. Left panel: WB using anti-p62 was imaged at 800 nm.   Right panel: the same WB using anti-GAPDH was imaged at 700 nm as a loading control.

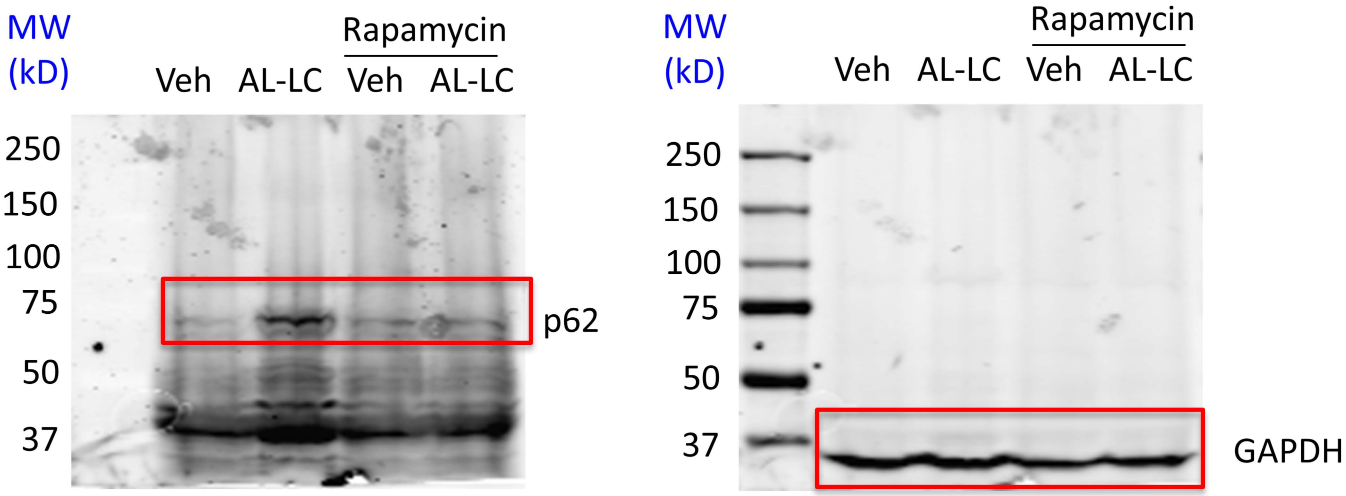

Source data for Figure 3F. Left panel: WB using anti-active caspase 3 was imaged at 700 nm. Right panel: the same WB using anti-β-actin was imaged at 800 nm as a loading control.

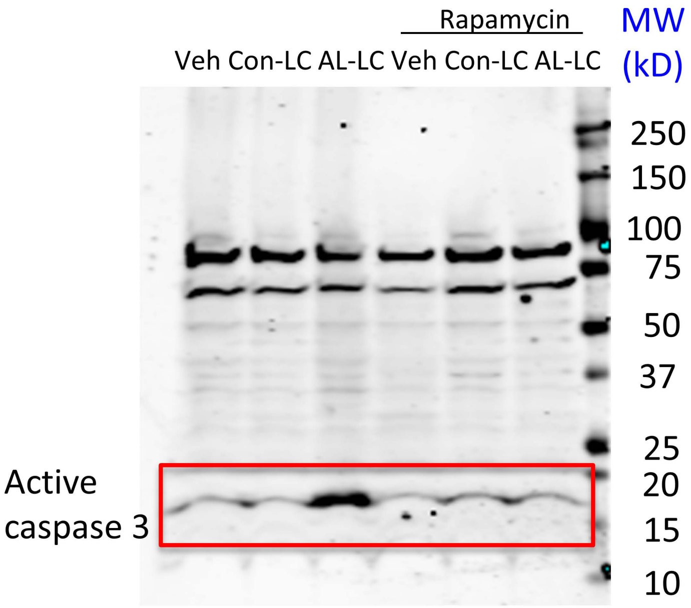

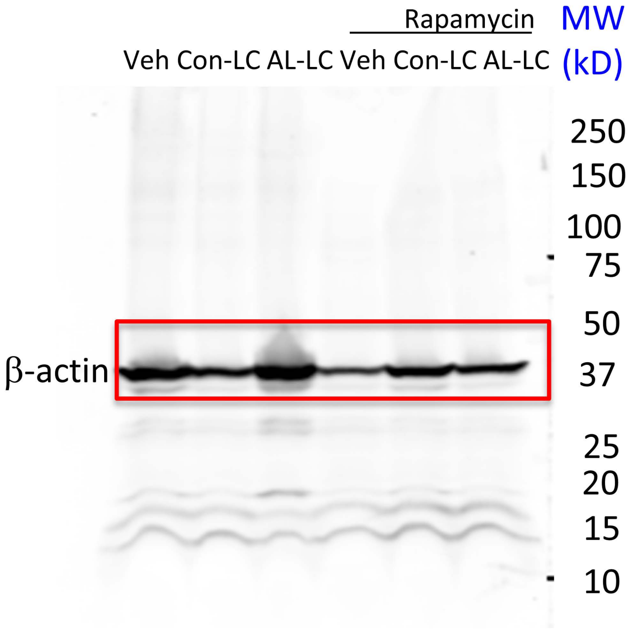

Supplement: Supplementary file 4 [file emmm0006-1493-sd4.pdf]
