# Supplementary material for: Lysosomal dysfunction and impaired autophagy underlie the pathogenesis of amyloidogenic light chain-mediated cardiotoxicity
Source: EMBO Mol Med. 2014 Oct 15;6(11):1493–507. doi: 10.15252/emmm.201404190 (PMC4237473; doi:10.15252/emmm.201404190)

**Source data for Figure 5B.** The red box indicates the cropped image used in the manuscript figure. Top panel: WB using anti-GFP was imaged at 800 nm. Bottom panel: the same WB using anti- GAPDH was imaged at 700 nm as a loading control.

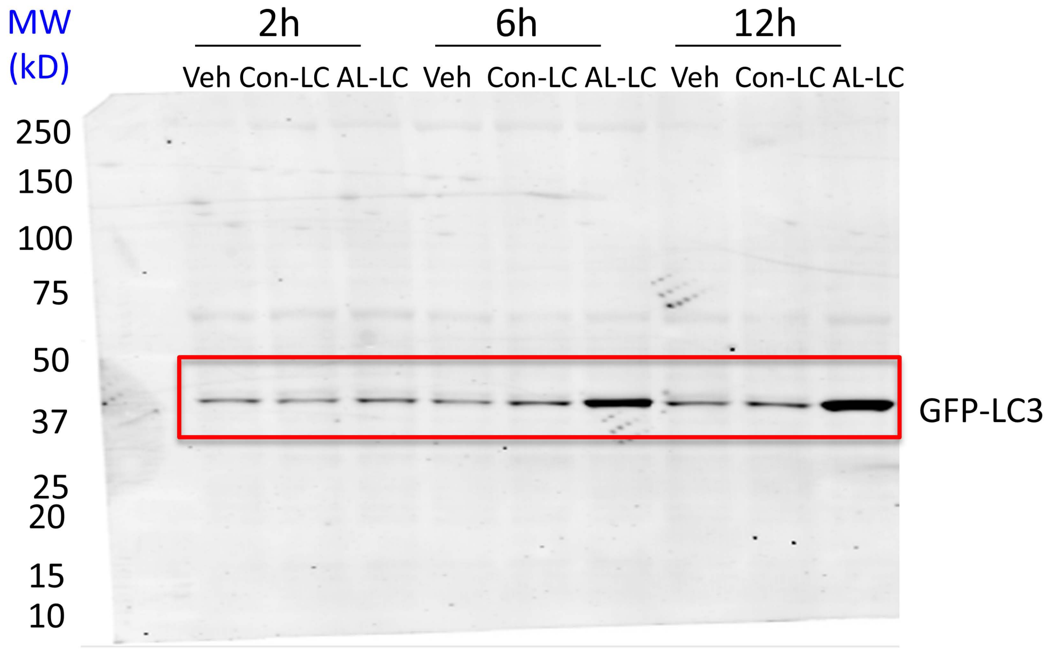


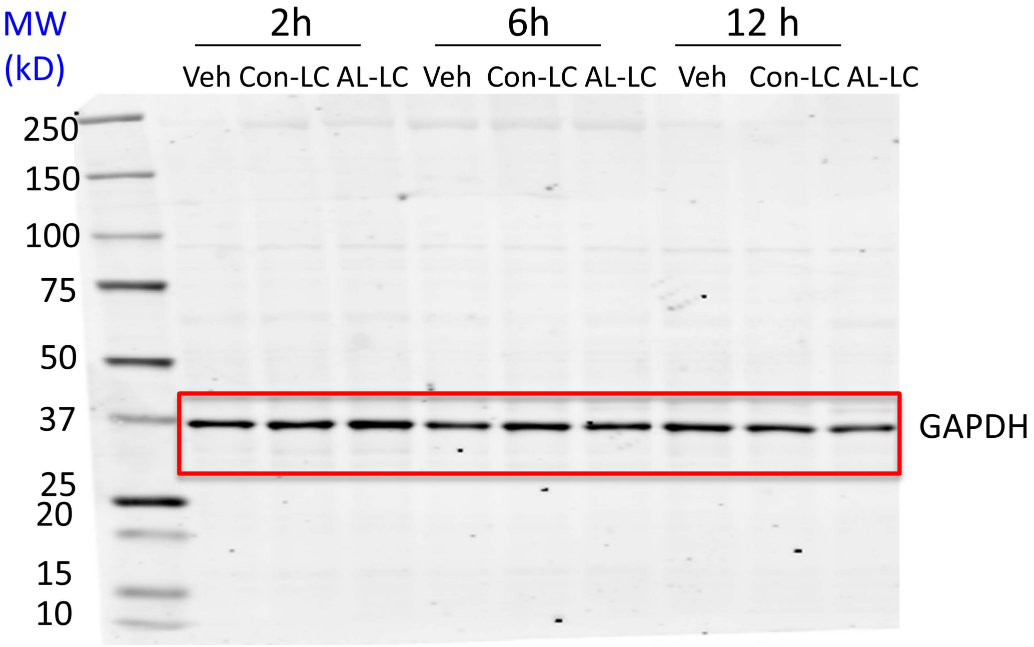

Supplement: Supplementary file 5 [file emmm0006-1493-sd5.pdf]
