# Supplementary material for: Lysosomal dysfunction and impaired autophagy underlie the pathogenesis of amyloidogenic light chain-mediated cardiotoxicity
Source: EMBO Mol Med. 2014 Oct 15;6(11):1493–507. doi: 10.15252/emmm.201404190 (PMC4237473; doi:10.15252/emmm.201404190)

Source Data for Figure 6B. The red box indicates the cropped image used in the manuscript figure. Top panel: WB using anti-LC3 was imaged at 700 nm. Bottom panel: the same WB using anti- β-actin was imaged at 800 nm as a loading control.

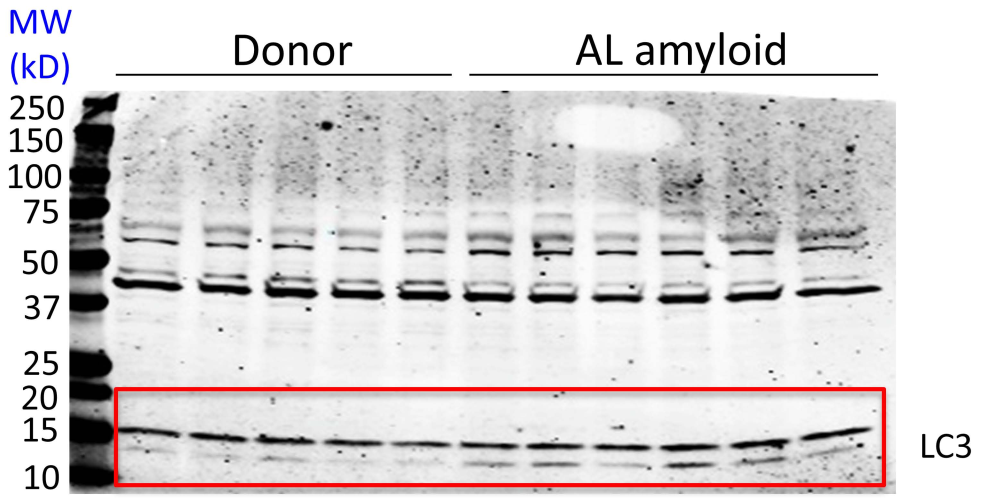

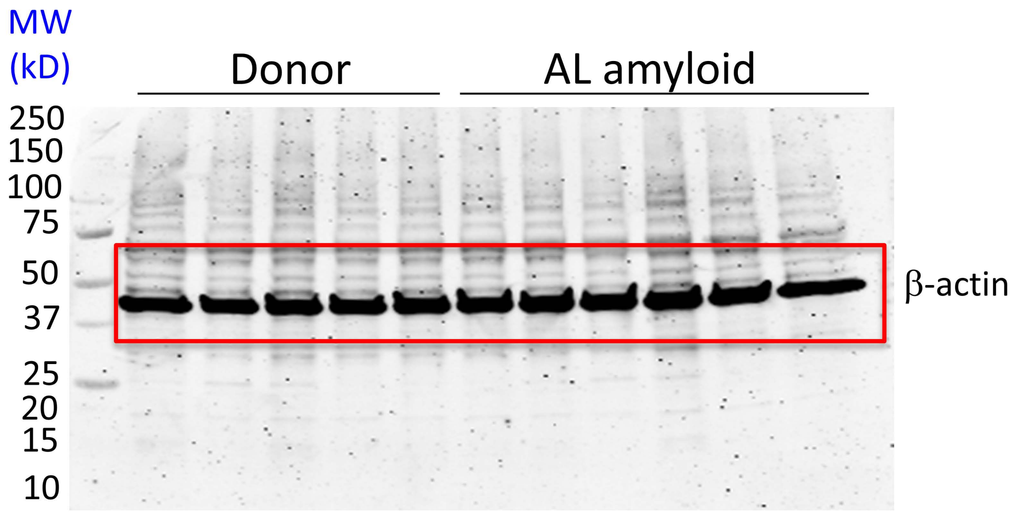

Source data for Figure 6C. Top panel: WB using anti-p62 was imaged at 800 nm. Bottom panel: the same WB using anti-GAPDH was imaged at 700 nm as a loading control.

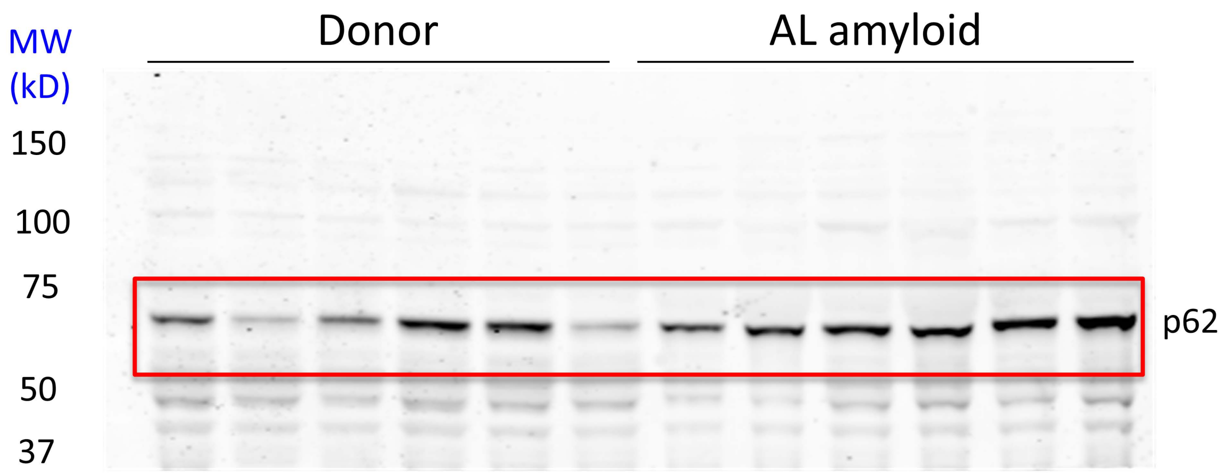

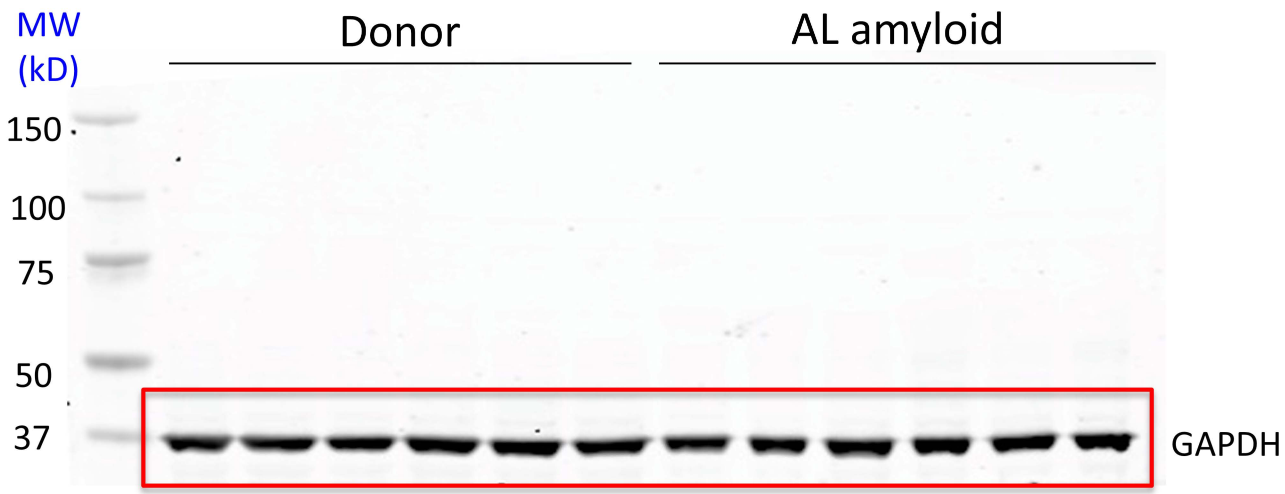

Source data for Figure 6D. Top panel: WB using anti-TFEB was imaged at 700 nm. A blocking peptide (right-most lane) was used to confirm the band selected was TFEB. Bottom panel: the same WB using anti-GAPDH was imaged at 800 nm as a loading control. Dashed line indicates cut in membrane for blocking peptide analysis.

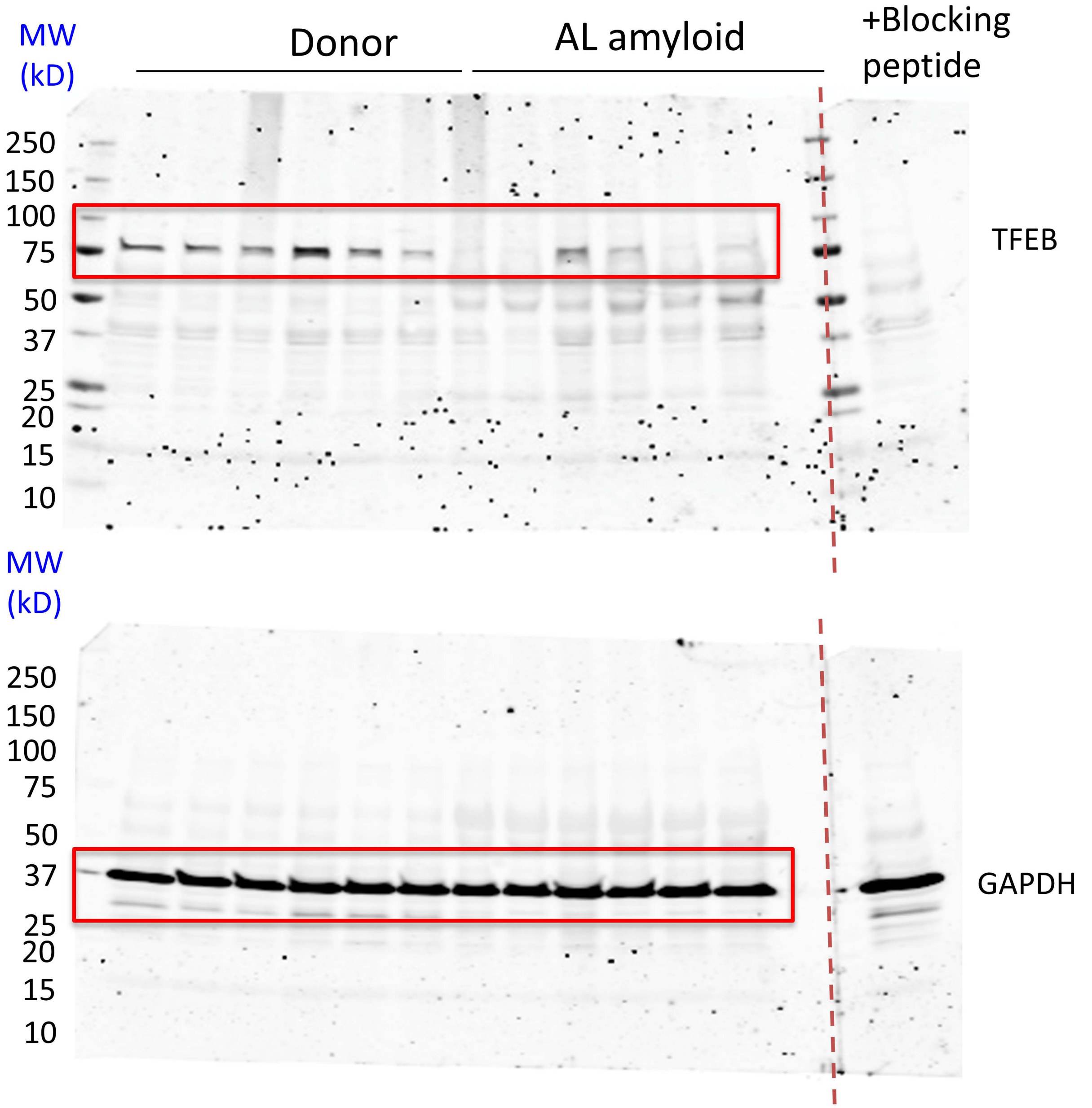

Supplement: Supplementary file 6 [file emmm0006-1493-sd6.pdf]
