# Supplementary material for: Lysosomal dysfunction and impaired autophagy underlie the pathogenesis of amyloidogenic light chain-mediated cardiotoxicity
Source: EMBO Mol Med. 2014 Oct 15;6(11):1493–507. doi: 10.15252/emmm.201404190 (PMC4237473; doi:10.15252/emmm.201404190)
Supplement: Supplementary file 7 [file emmm0006-1493-sd7.pdf]

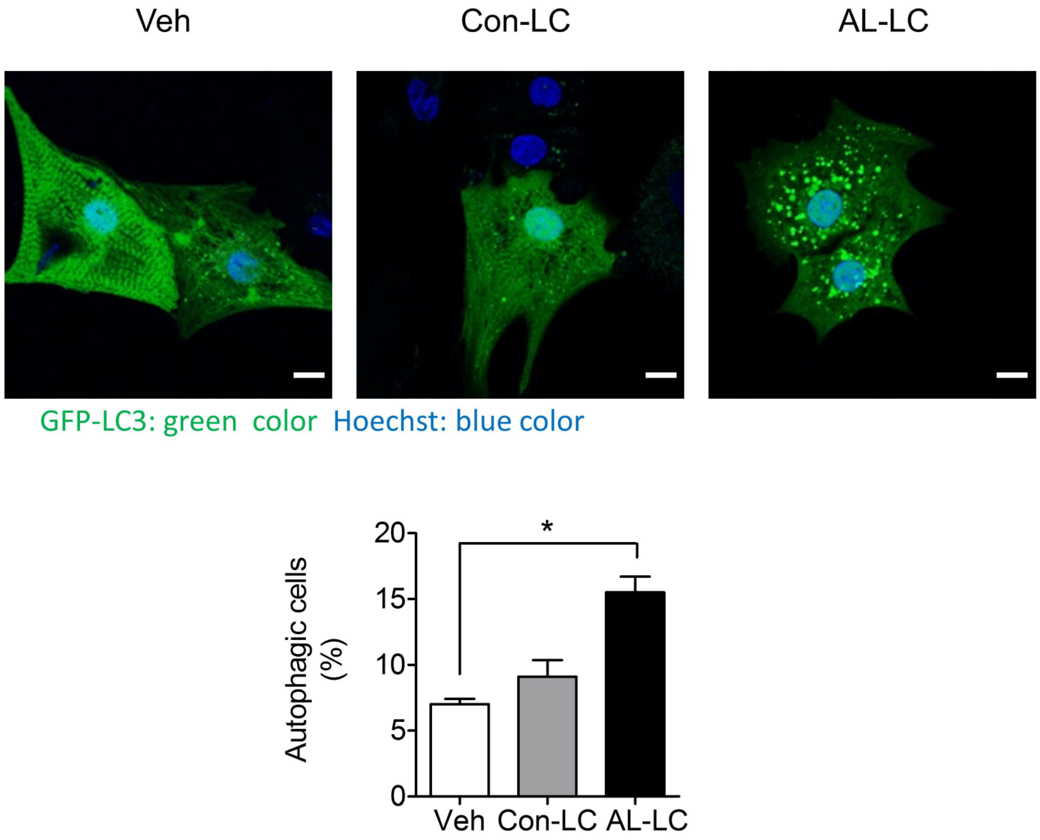

Figure S1. Autophagosome visualization in cardiomyocytes following AL-LC exposure

Autophagosome formation was visualized using GFP-LC3 expression in cardiomyocytes exposed to either Veh, Con-LC or AL-LC for 24 hours. AL-LC cardiomyocytes exhibited increased autophagosome formation compared to controls. Scale bar=10 μm. N=6-7.
* $p=2.2\times10^{-4}$ between AL-LC and Veh group.

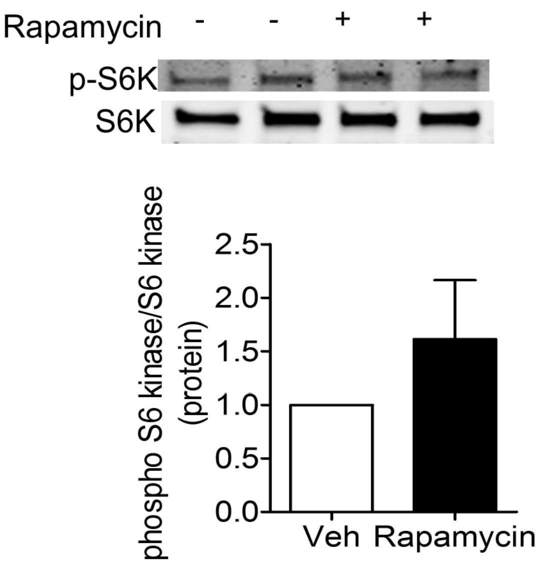

Figure S2. Rapamycin (10 nM) does not inhibit S6 kinase (S6K)

S6K activation is not altered following treatment with Rapamycin (10 nM) as shown by immunoblot analysis (above) and quantitative summary of S6K activation (below).

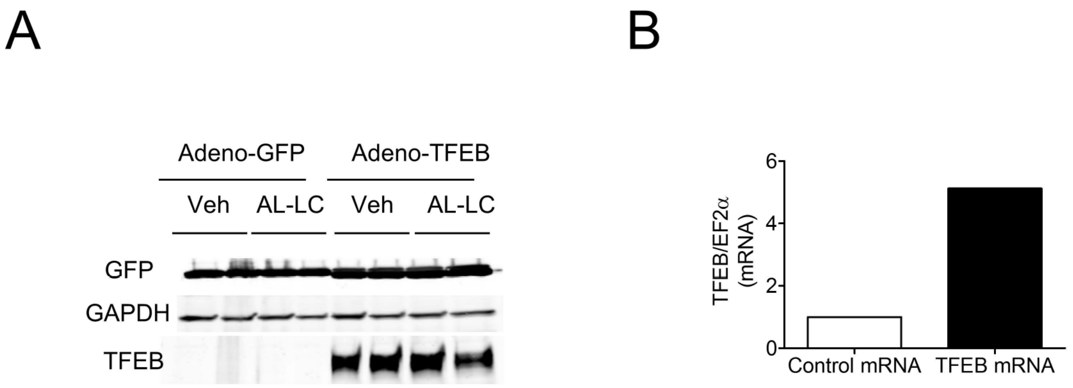

Figure S3. Overexpression of TFEB in cardiomyocytes and zebrafish.

A. Following adenoviral infection of adult cardiomyocytes, expression level of TFEB was measured by immunoblot. GFP and GAPDH serve as controls.
B. TFEB expression was validated by real-time PCR following transient overexpression of TFEB in zebrafish via mRNA injection at the single cell stage. Reverse TFEB mRNA was used as a control. N=2 independent replicates replicates, each replicate consisting of 15 individual embryos.

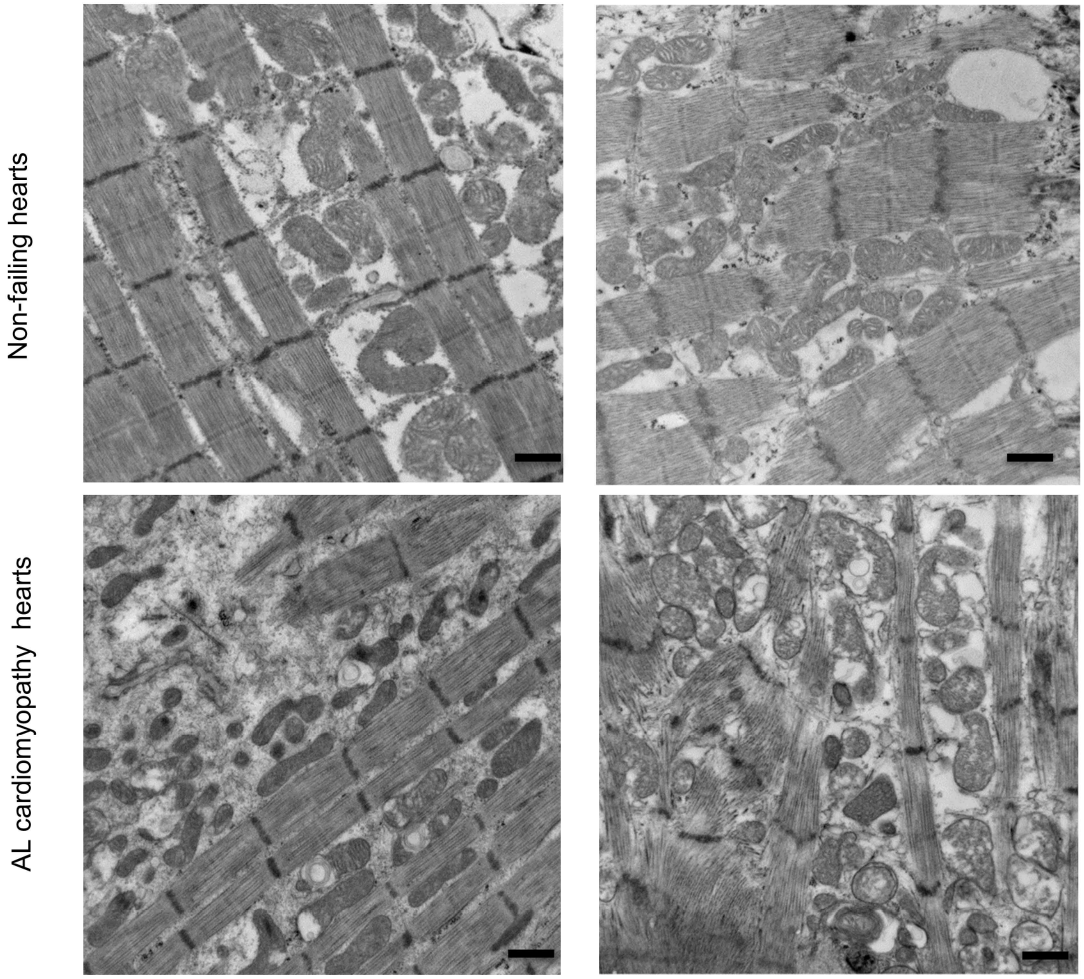

Figure S4. EM images of human heart tissue

Diffuse mitochondrial abnormalities were observed in heart tissues from AL amyloid cardiomyopathy patients relative to control, non-failing human hearts. Scale bar=500 nm.

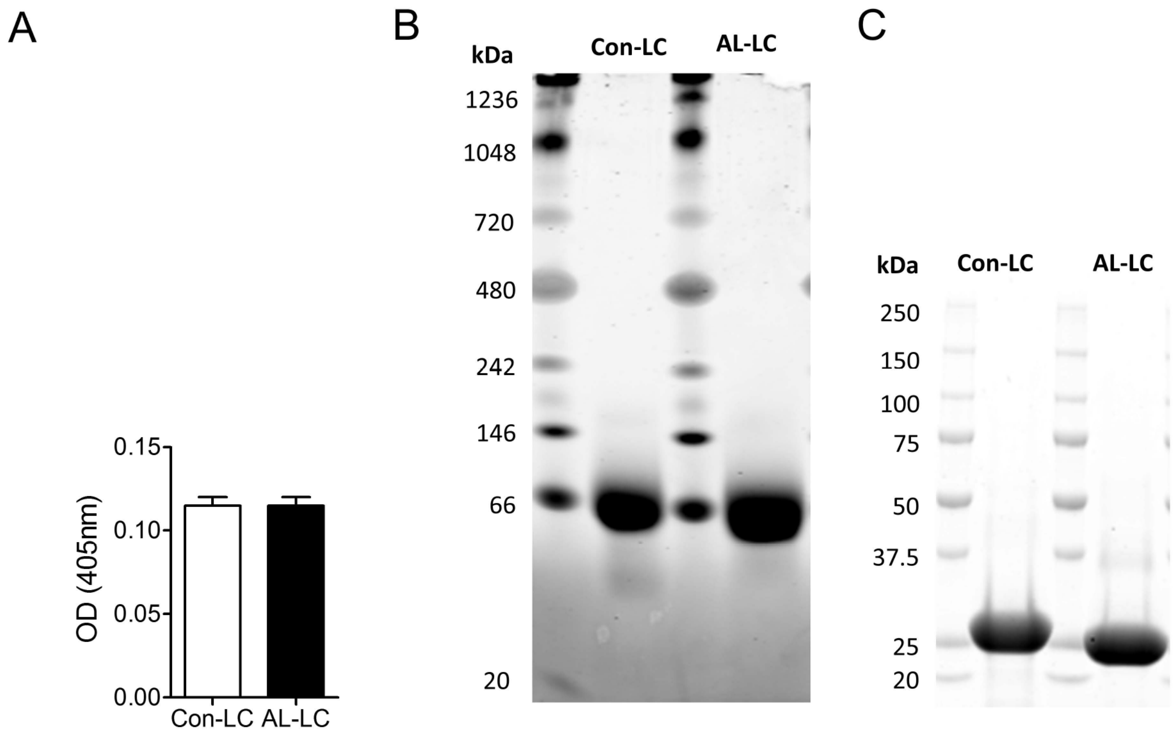

Figure S5. Biochemical characterization of light chains

A. Turbidity assay to assess solubility of Con-LC (20 μg/ml) and AL-LC (20 μg/ml). Coomassie blue stain of a gel run under non-reducing conditions (**B**) and reducing conditions (**C**).

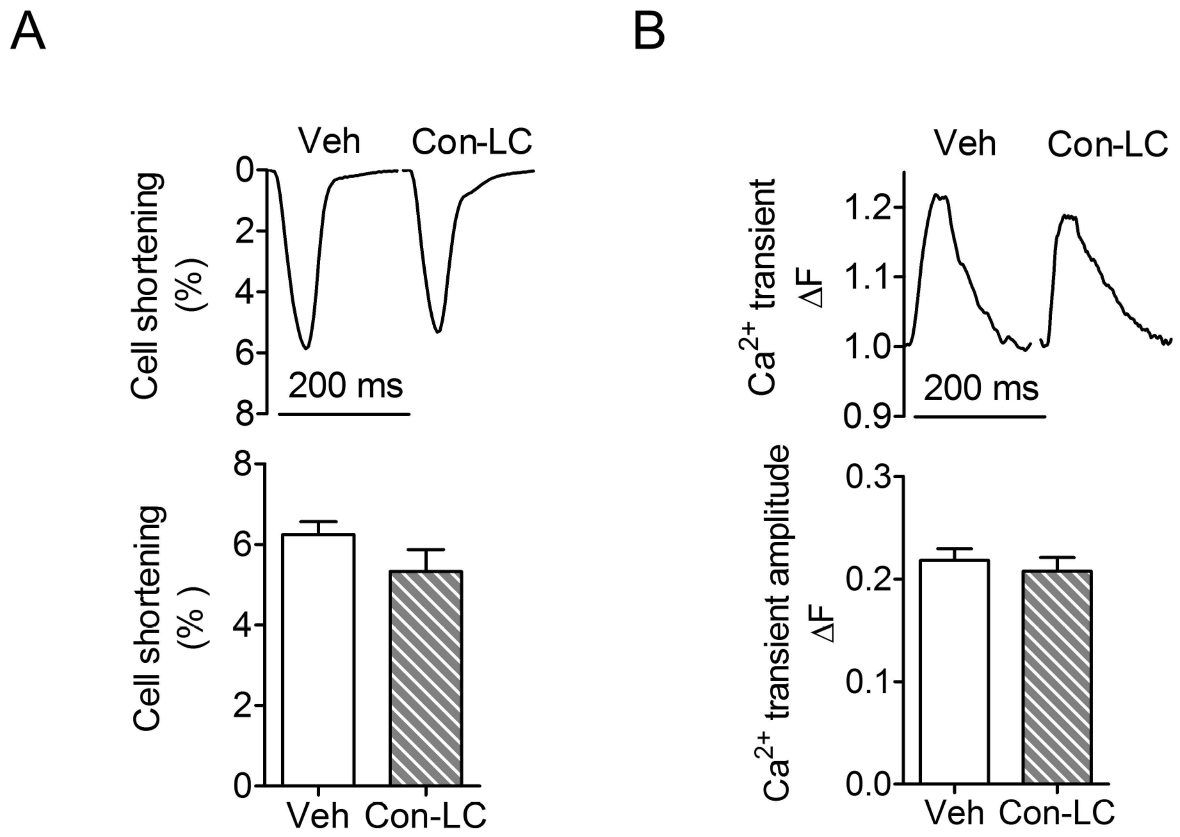

Figure S6. High concentration of Con-LC does not affect contractile function and calcium homeostasis in cardiomyocytes

Isolated adult cardiomyocytes were exposed to100 μg/ml Con-LC for 24 hours, and **A.** contractile function and **B.** intracellular calcium transient were measured. Representative tracings are shown in the top panel, and quantitative analysis for percent cell shortening or calcium transient amplitude is shown below. N=3 biological replicates with 6-10 cells averaged per replicate.

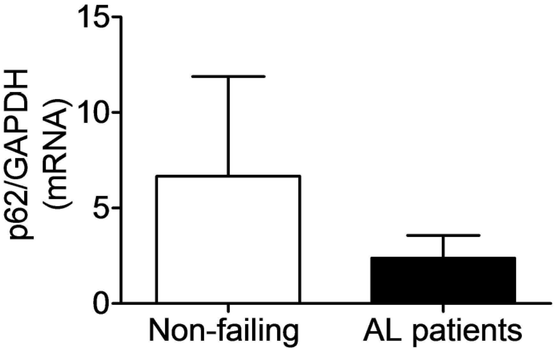

Figure S7. p62 mRNA expression in human heart tissue

Expression of p62 mRNA relative to GAPDH in human heart tissues was measured from non-failing control hearts and hearts obtained from AL amyloid cardiomyopathy patients (AL patients) using real-time PCR. N=6.

Table S1: Human amyloidogenic light chain proteins used in study

	**Diagnosis**	**LC isotype**	**Gender**	**Amyloid organ involvement**
Con-LC	MM	κ1	M	None
AL-LC1	AL	λ3	M	cardiac predominance
AL-LC2	AL	λ2	M	cardiac predominance
AL-LC3	AL	λ1	M	cardiac involvement with soft tissue predominance

Abbreviations: control light chain proteins (Con-LC); amyloidogenic light chain proteins (AL-LC), multiple myeloma (MM), AL primary amyloidosis (AL), male (M), kappa light chain isotype (κ), lambda light chain isotype (λ). For AL-LC1, AL-LC2, AL-LC3, multiple tissue involvement was observed, with tissue predominance noted.

Table S2: Patient demographic information

	**Non-failing hearts**	**AL amyloid cardiomyopathy hearts**
Number	2/4 (M/F)	4/2 (M/F)
Age (years)	63.5±6.2	51±4.5
Heart weight (grams)	363±77	521±43

Abbreviations: AL amyloidosis (AL), female (F), male (M)

Supplementary Methods

Turbidity assay

Con-LC or AL-LC was dissolved in water at the concentration of 20 μg/ml. The turbidity of the solution was determined spectrophotometrically at 405 nm using a plate reader (SpectraMax, Molecular Devices) (Dickens & Franz, 2010).

Characterization of human light chain proteins

Native gel electrophoresis was performed to characterize the non-reducing conditions of light chain protein (Park et al, 2012). Briefly, a total 20 μg of either Con-LC or AL-LC was diluted in the NuPAGE LDS sample buffer (Invitrogen). For non-reducing conditions, Con-LC or AL-LC was boiled for 5 minutes and then added into a NativePAGE™ Bis Tris gel(4-16%) for electrophoresis at a constant voltage of 150v. NativeMark™ Unstained Protein Standard (Invitrogen) was used as a marker. For reducing conditions, a total 20 μg of either Con-LC or AL-LC was diluted in Laemmli sample buffer (Bio-rad) supplemented with β-mercaptoethanol. Samples were boiled for 5 minutes, and then gel electrophoresis was performed using a Pager Gold precast gel (4-20%) (Lonza). Coomassie blue staining was performed to visualize protein.

Autophagosome visualization

To visualize autophagosomes, neonatal cardiomyocytes were cultured in glass-bottomed petri-dishes (Mat-tek). Cardiomyocytes were infected with GFP-LC3 adenovirus (Mizushima et al, 2010) (a gift from Drs. Noboru Mizushima and Junichi Sadoshima). Twenty-four hours following infection, cells were exposured to either Vehicle or AL-LC for 48 hours. Cells were then incubated with Hoechst dye (2 μg/ml) for 30 minutes to visualize nuclei and autophagosomes. Cells were imaged using a LSM700 confocal microscopy (Zeiss). Cells with more than 5 visible autophagosomes were included in analysis as an autophagic cell.

S6 kinase western blotting analysis

Adult cardiomyocytes were treated with 10 nM rapamycin for 24 hours. Following wash with 1X PBS, cells were harvested using a cell lifter. Protein homogenate was harvested from lysed cells and subjected to immunoblotting for phospho-S6 kinase and total S6 kinase (antibody information provided below).

PCR for p62 mRNA expression in human heart tissue

Total RNA was isolated from human tissues using Trizol (Invitrogen) extraction as previously described (Shi et al, 2010). DNAase treatment and reverse transcription were performed to remove residual DNA contamination and to synthesize cDNA. Real-time PCR was performed to determine p62 expression levels using the following primers:
p62 forward primer- GGCTGATCCCCGGCTGATTGAG; reverse primer- ACTTGGCCACAGCACTGTCACAA

Additional information for all source data files

Western blot (WB) scans were obtained using a LI-COR Odyssey infrared scanner. For each WB image, the red box indicates the cropped image used in the manuscript figure; full uncropped WB images are provided in the accompanying respective source data files. All antibody sources and dilutions are defined in the table below. GAPDH or β-actin was used as loading controls. Fluorescent Molecular Weight (MW) marker was only visualized at 700nm.

Antibody	Source	Dilution
GAPDH	R&D	1:2000
β-actin	Sigma	1:2000
LC-3	MBL	1:1000
p62	Abnova	1:1000
Active caspase3	Abcam	1:500
GFP	Cell signaling	1:1000
S6Kinase	Cell signaling	1:500
Phospho-S6Kinase	Cell signaling	1:500
TFEB	Santa Cruz Biotech	1:500

References

Dickens MG, Franz KJ (2010) A prochelator activated by hydrogen peroxide prevents metal-induced amyloid Beta aggregation. Chembiochem 11**:** 59-62

Mizushima N, Yoshimori T, Levine B (2010) Methods in mammalian autophagy research. Cell 140**:** 313-326

Park YN, Zhao X, Norton M, Taylor JP, Eisenberg E, Greene LE (2012) Huntingtin fragments and SOD1 mutants form soluble oligomers in the cell. PLoS One 7**:** e40329

Shi J, Guan J, Jiang B, Brenner DA, Del Monte F, Ward JE, Connors LH, Sawyer DB, Semigran MJ, Macgillivray TE et al (2010) Amyloidogenic light chains induce cardiomyocyte contractile dysfunction and apoptosis via a non-canonical p38alpha MAPK pathway. Proc Natl Acad Sci U S A 107**:** 4188-4193
