# Supplementary material for: Lysosomal dysfunction and impaired autophagy underlie the pathogenesis of amyloidogenic light chain-mediated cardiotoxicity
Source: EMBO Mol Med. 2014 Oct 15;6(11):1493–507. doi: 10.15252/emmm.201404190 (PMC4237473; doi:10.15252/emmm.201404190)

Source data for Figure S2. The red box indicates the cropped image used in the manuscript figure. Left panel: WB using anti-phospho S6K was imaged at 800 nm. Right panel: the same WB using anti-S6K was imaged at 700 nm.

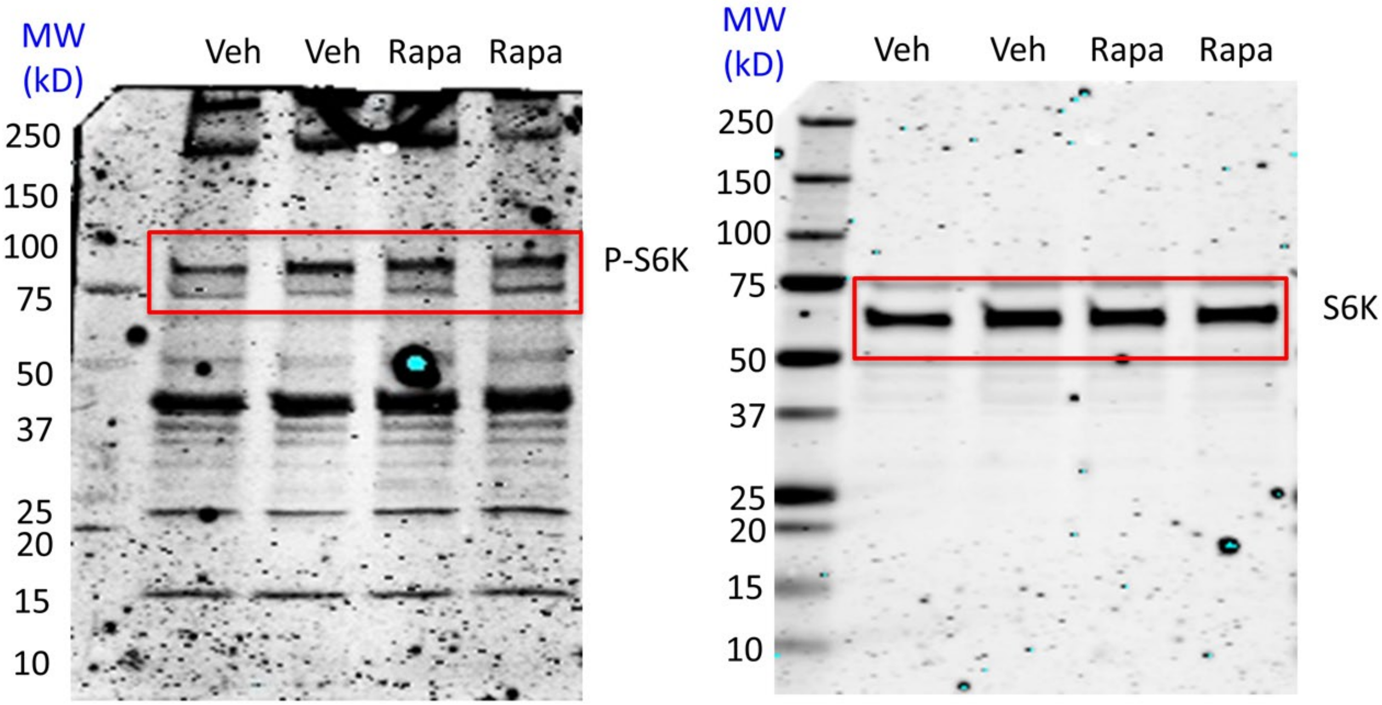

Supplement: Supplementary file 8 [file emmm0006-1493-sd8.pdf]
